# Ecological niche model transferability of the white star apple (*Chrysophyllum albidum* G. Don) in the context of climate and global changes

**DOI:** 10.1038/s41598-023-29048-3

**Published:** 2023-02-10

**Authors:** Jean Cossi Ganglo

**Affiliations:** Laboratory of Forest Sciences, Faculty of Agricultural Sciences, University of Abomey-Calavi (Benin), Abomey-Calavi, Benin

**Keywords:** Computational biology and bioinformatics, Ecology, Plant sciences, Climate sciences, Ecology, Environmental sciences

## Abstract

*Chrysophyllum albidum* is a forest food tree species of the Sapotaceae family bearing large berries of nutrition, sanitary, and commercial value in many African countries. Because of its socioeconomic importance, *C. albidum* is threatened at least by human pressure. However, we do not know to what extent climate change can impact its distribution or whether it is possible to introduce the species in other tropical regions. To resolve our concerns, we decided to model the spatial distribution of the species. We then used the SDM package for data modeling in R to compare the predictive performances of algorithms among the most commonly used: three machine learning algorithms (MaxEnt, boosted regression trees, and random forests) and three regression algorithms (generalized linear model, generalized additive models, and multivariate adaptive regression spline). We performed model transfers in tropical Asia and Latin America. At the scale of Africa, predictions with respect to Maxent under Africlim (scenarios RCP 4.5 and RCP 8.5, horizon 2055) and MIROCES2L (scenarios SSP245 and SSP585, horizon 2060) showed that the suitable areas of *C. albidum*, within threshold values of the most contributing variables to the models, will extend mostly in West, East, Central, and Southern Africa as well as in East Madagascar. As opposed to Maxent, in Africa, the predictions for the future of BRT and RF were unrealistic with respect to the known ecology of *C. albidum.* All the algorithms except Maxent (for tropical Asia only), were consistent in predicting a successful introduction of *C. albidum* in Latin America and tropical Asia, both at present and in the future. We therefore recommend the introduction and cultivation of *Chrysophyllum albidum* in the predicted suitable areas of Latin America and tropical Asia, along with vegetation inventories in order to discover likely, sister or vicarious species of *Chrysophyllum albidum* that can be new to Science. Africlim is more successful than MIROCES2L in predicting realistic suitable areas of *Chrysophyllum albidum* in Africa. We therefore recommend to the authors of Africlim an update of Africlim models to comply with the sixth Assessment Report (AR6) of IPCC.

## Introduction

Biodiversity and nature’s contributions provide many goods and services indispensable to the survival of populations such as food, crops, woods, regulation of climate change through carbon storage in vegetation biomass, spiritual enrichment, recreation and tourism etc.^1,2^. Despite its utmost importance, biodiversity is subjected to serious threats that undermine human well-being and impede the survival of humanity^1–4^. Biodiversity loss is mostly caused by human-induced drivers, including overexploitation, pollution, invasive alien species, habitat fragmentation, agricultural expansion, climate change, and poaching, as well as natural drivers such as disease and pest outbreaks^1,3–7^.

Across regions, climate change is resulting in increases in temperature and sea level, heat waves, and changes in rainfall patterns such as heavy precipitation and droughts^6^. All this affects the ecological niches of living organisms and then exacerbates the other drivers of biodiversity loss^1^. Mitigating the impacts of climate change on biodiversity therefore needs to be approached seriously. This is why many studies have been conducted worldwide to contribute to biodiversity conservation in the context of climate and global changes. Most of them used species distribution models (SDMs). The SDMs (in geographic space), also known as ecological niche models (ENMs) (in environmental space), explore the relationship between species occurrences and environmental variables to predict the distribution of species at present and in the future and therefore support policy decisions on biodiversity conservation and sustainable uses^8,9^. SDMs are widely used to answer many research questions and provide guidance for biodiversity conservation in various fields of investigation such as the discovery of new species^10^; identification of potential invasion hotspots of alien species^11,12^; predictions of range-shifting species and pest risks to useful plants^13,14^; identification and characterization of suitable areas of useful species and contribution to their conservation^15–35^; identification of spatial pattern of species abundance and richness^36–38^; understanding the species richness patterns in response to climate change^39^; identification and characterization of fragmentation risks of species habitats^40^; public health, risk maps and risk prevention of vector-borne diseases like Ebola fever, Lassa fever, dengue, African swine fever etc.^41–45^; model transfers to guide species relocations, introductions and reintroductions; promotion of species-habitat conservation and forecasting areas vulnerable to invasion ; develop plans for habitat management, quantify potential of unoccupied habitat, facilitate long-term species persistence^46–50^.

Using SDMs, Brychkova et al.^27^ investigated the impacts of climate change on three forage grasses useful for Ethiopian dairy systems. They determined areas of geographic suitability for each species and calculated their ability to meet predicted dry matter demand under hypotheses of livestock intensification and land availability. They found out that by 2050, Buffel grass (*Cenchrus ciliaris*) is likely to be negatively affected by climate change in regions like Tigray, while Rhodes grass (*Chloris gayana*) and Napier grass (*Cenchrus purpureus*) may have improved suitability areas under future climates. From their results, feed demands could be met by production of these forage grasses under current and future climates if the current land availability and herd composition are maintained.

Karami et al.^31^ determined the distributional patterns for a semi-endemic medicinal plant species, *Nepeta glomerulosa,* growing in southwestern and central Asia using Maxent method under two Shared Socioeconomic Pathways scenarios (SSP2-4.5 and SSP5-8.5) of climate change for 2060. The predictions in the future indicated that suitable areas for the species will increase in general although the types and degrees of these changes differ among areas.

Lyam et al.^51^ investigated the major climatic factors responsible for the geographical distribution of *Chrysophyllum albidum* in South-West part of Nigeria using Maxent. They found out that the main variables that contributed towards predicting the species distribution were temperature seasonality, minimum temperature of coldest month and mean temperature of driest quarter. The distribution model confirmed the wide distribution of *C. albidum* in South-West Nigeria.

*Chrysophyllum albidum* is a forest food tree species of the Sapotaceae family. It is found in lowland rain forests and is widely distributed in West, Central and East Africa^52–54^. It grows in South Benin, which has a subequatorial climate, on ferallitic soils^55^. Its fruits are large berries of nutritional, sanitary, and commercial value in many African countries. Many use categories were recorded for *C. albidum* among which food purpose was dominant, especially the fleshy pulp of the fruits, which is largely eaten by local people^53,56,57^. The authors pointed out that different parts of the species, especially the bark, leaves, seeds, and fruits, are used in folk medicine for treatments of malaria, sterility, sexual asthenia, asthma, intestinal worms, hemorrhoid, cough, icterus, yellow fever, avitaminosis, dental decay, etc. Furthermore, the leaf and fruit essential oil of *Chrysophyllum albidum* have potential use in cosmetic and pharmaceutical industries as preservative and pharmaceutical agents^58^.

Despite its socioeconomic importance, *C. albidum* is threatened and neglected by people. Indeed, with respect to regeneration and improvement, *C. albidum* is considered a neglected species^53^*.* In Nigeria, the species is listed as endangered, prone to extinction^59^, while in Benin, it is considered vulnerable^56^.

Real time data indicate that global greenhouse gas emissions continue to increase in 2022 and this will exacerbate climate change so that the Global Mean Surface Temperature is estimated at 1.15 ± 0.13 °C warmer than the pre-industrial baseline (1850–1900)^6^. Furthermore, the predictions of increase in temperature up to 02 °C at least above the current level of temperature by all climatic models at horizon 2055–2060 in Africa, Latin America, and Asia^60,61^, will more exacerbate the effect of climate change on species habitats including the ecological niche of *Chrysophyllum albidum*.

Due to the high socioeconomic, health, nutrition, and commercial value of the species and its vulnerability to human pressure and likely climate change, we decided to investigate it. The research questions that guided our investigation were as follows: 1) what are the favorable areas where to grow *C. albidum* in Africa in the context of human pressure and climate change? 2) Does a regional circulation model such as Africlim better predict the spatial distribution of the species than a general circulation model such as MIROCES2L? 3) Can the species successfully be introduced in tropical Asia and Latin America to become a pan-tropical forest food species of interest for the populations of America and Asia? Our findings can guide climate adaptation and landscape management strategies for the conservation of *C. albidum* and its dissemination and promotion worldwide in the tropics.

## Material and methods

### Species data

**Figure 1:**
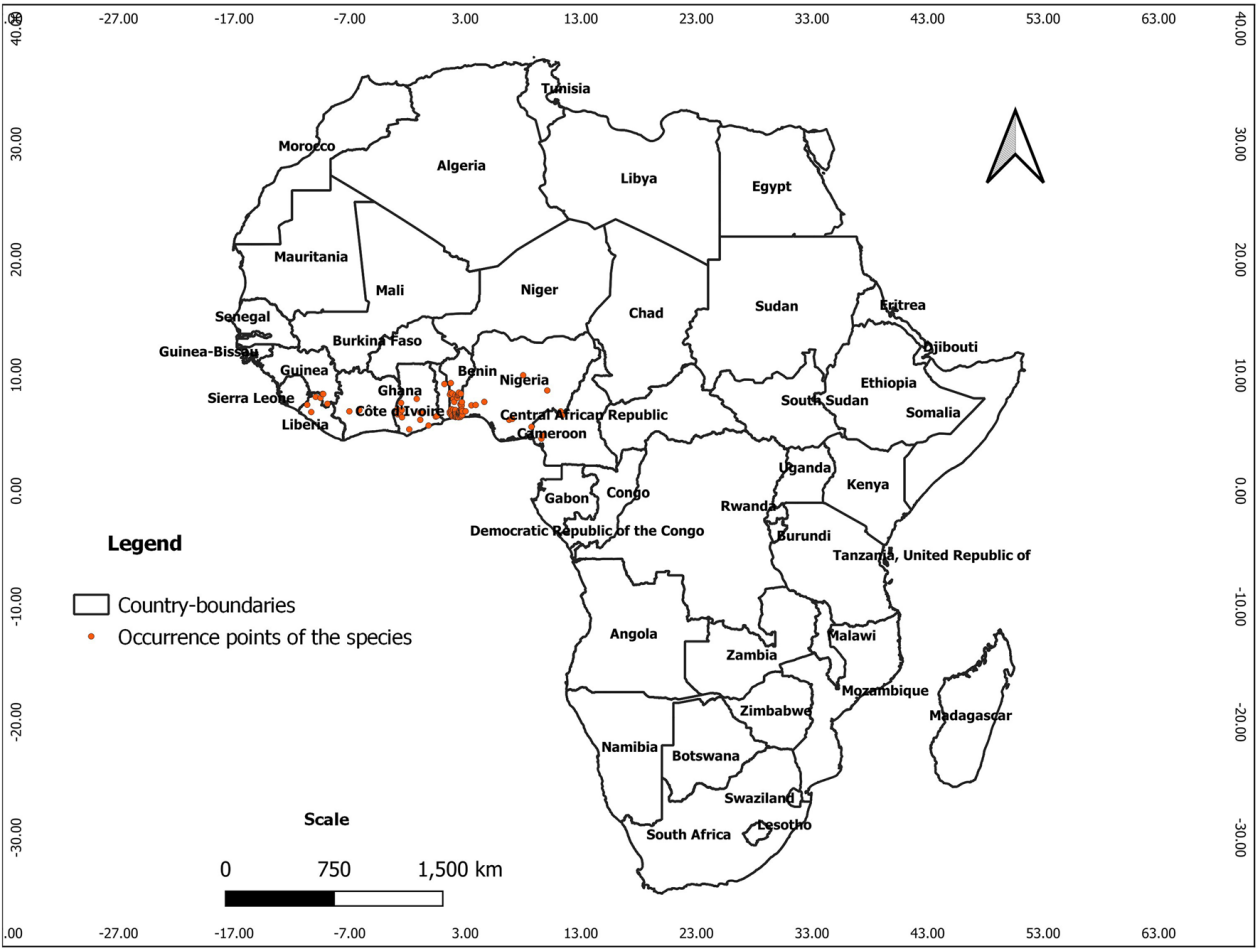
Spatial distribution of the occurrence points of *C. albidum* across Africa (the map was generated using QGIS 3.18.1 (http://qgis.osgeo.org) and WGS 84 as Coordinate Reference System). Source of occurrence points: GBIF.org (1 March 2021) GBIF Occurrence Download https://doi.org/10.15468/dl.pg7stw.

We explored many online repositories to download occurrence data. They are databases of the Global Biodiversity Information Facility (GBIF, www.gbif.org), Atlas of living Australia (www.ala.org.au), iNaturalist (www.inaturalist.org), and speciesLink (www.splink.org.br). We cleaned the data, removing duplicates, data without coordinates, and managed and introduced specimens. *Chrysophyllum albidum* is a terrestrial species, and we therefore also removed data falling in the ocean. To address problems associated with spatial sampling biases of the occurrence records, we used the spThin R package version 0.2.0^62^ for spatial thinning of the records at a minimum distance of 5.0 km to comply with the spatial resolution of 2.5 arc minutes of the environmental data used in the models. The dataset used in the model calibration is displayed in Fig. 1.

### Predictor data

Data resolution influences the quality of models, their predictions, and their transferability^49,63^. Poorly resolved predictors are unlikely to match the ecology of the species^64^. Therefore, taking into account the spatial density of occurrence data available on *C. albidum*, we used predictor data at a resolution of 2.5 min arc. They are made of 17 bioclimatic variables of the present, downloaded at a spatial resolution of 2.5 min arc on the WorldClim site^65^. We also used data on human population density as a proxy for human pressure, downloaded at the same resolution, from the Socio-Economic Data and Application Center (SEDAC)^66^. Future bioclimatic environmental data for projection purposes in Africa were downloaded at the Africlim site^60^. The ensemble climatic models^60^ were therefore used at a resolution of 2.5 min arc. We used the Africlim data for future projection in Africa because it is advantageous over the general circulation models of WorldClim and is available for Africa. Indeed, as regional models, they were downscaled to fit African realities (high density of population living in coastal areas, marked mountainous topography, etc.)^60^. Future bioclimatic data for projections to tropical Asia and tropical America were downloaded from the WorldClim site. We therefore used the general circulation Model for Interdisciplinary Research on Climate, Earth System version 2 for long-term simulations (MIROCES2L)^61^ under shared socioeconomic pathways 2 (SSP2) and 5 (SSP5), which approximately correspond to the RCP4.5 and RCP8.5 scenarios, respectively^67^. MIROCES2L is an improvement of the previous Earth System Models (ESM) (MIROC 5, MIROC 5.2, and MIROC6)^61^. Indeed, according to those authors, this new model includes a terrestrial biogeochemical component with explicit carbon–nitrogen interactions that enabled us to account for the change in land carbon fluxes; the new model also includes an updated ocean biogeochemical component to better describe the relationship between oceanic primary productivity and multiple nutrient limitations^61^. We addressed the problem of collinearity between environmental variables. Indeed, collinearity can induce bias in parameter estimation by inflating variance of regression parameters and therefore misleads in the choice of the relevant predictors of models^68,69^. To account for this, we extracted the values of the predictors at occurrence points and ran the variance inflation factor (VIF) function in the SDM package^69^. We therefore retained the less correlated variables in the models. Furthermore, preliminary running of models helped us identify among the less correlated variables the most contributing ones to the models.

### SDM package, model fitting, and evaluation

We used the SDM package^69^ for data modeling purposes in R^70^. It offered us the facilities needed to compare the predictive performances of several algorithms/modelling methods and to achieve the desired ensemble models. To compare algorithms and choose the best ones, among the most common, we used three machine learning algorithms: MaxEnt^71^, boosted regression trees (BRT)^72^, random forests (RF)^73^; and three regression algorithms: generalized linear model (GLM)^74^, generalized additive models (GAMs)^75^, and multivariate adaptive regression spline (MARS)^76^. In model calibration, the default settings^69^ were used. However, when creating the SDM object data, we generated 10,000 random background points for both types of algorithms. The cross validation method with 2 replications, making a total of 10 replications, was applied to run the models. The predictive performances of the models generated were measured through their discrimination capacities and reliabilities using different statistics, namely, the area under the curve (AUC) of the receiver-operating characteristics plot^8,9^, the true skill statistics (TSS)^77^, the point biserial correlation (COR), and the proportion of explained deviance (Deviance)^8^. Ensemble models were generated using the threshold that maximized the TSS. This threshold was shown to produce the most accurate predictions^78^. That threshold was also used to achieve binary classification of the outputs of the models in QGIS 3.18.1^79^.

In order to appraise the effect of climate change on the contributing variables to the models, we used the tool “Random points inside polygons” of the function “Vector creation” of QGIS 3.18.1^79^ to add 10,000 to 100,000 random points in Latin America, tropical Asia, and Africa and thereafter, we used the “extract” function of the Raster package in R^70^ to extract the values of the variables retained in the models at the random points set in the study areas. Their mean values and standard deviation (sd) were then calculated to appreciate the effect of climate change on their evolution and their contribution to the suitability of the habitat of *Chrysophyllum albidum* with respect to the different climatic models and scenarios used.

## Results

The results are presented here, first with predictions at present to appraise the performances of the different algorithms, and then the projections in the future are presented with outputs of the most efficient algorithms with respect to the known ecology of the species. The differences noted between the outputs of MIROCES2L and those of Africlim are presented and discussed as well. To account for the clarity of the text, some figures are presented in Appendices as electronic supplementary materials online.

### Selected variables and model validation

**Figure 2:**
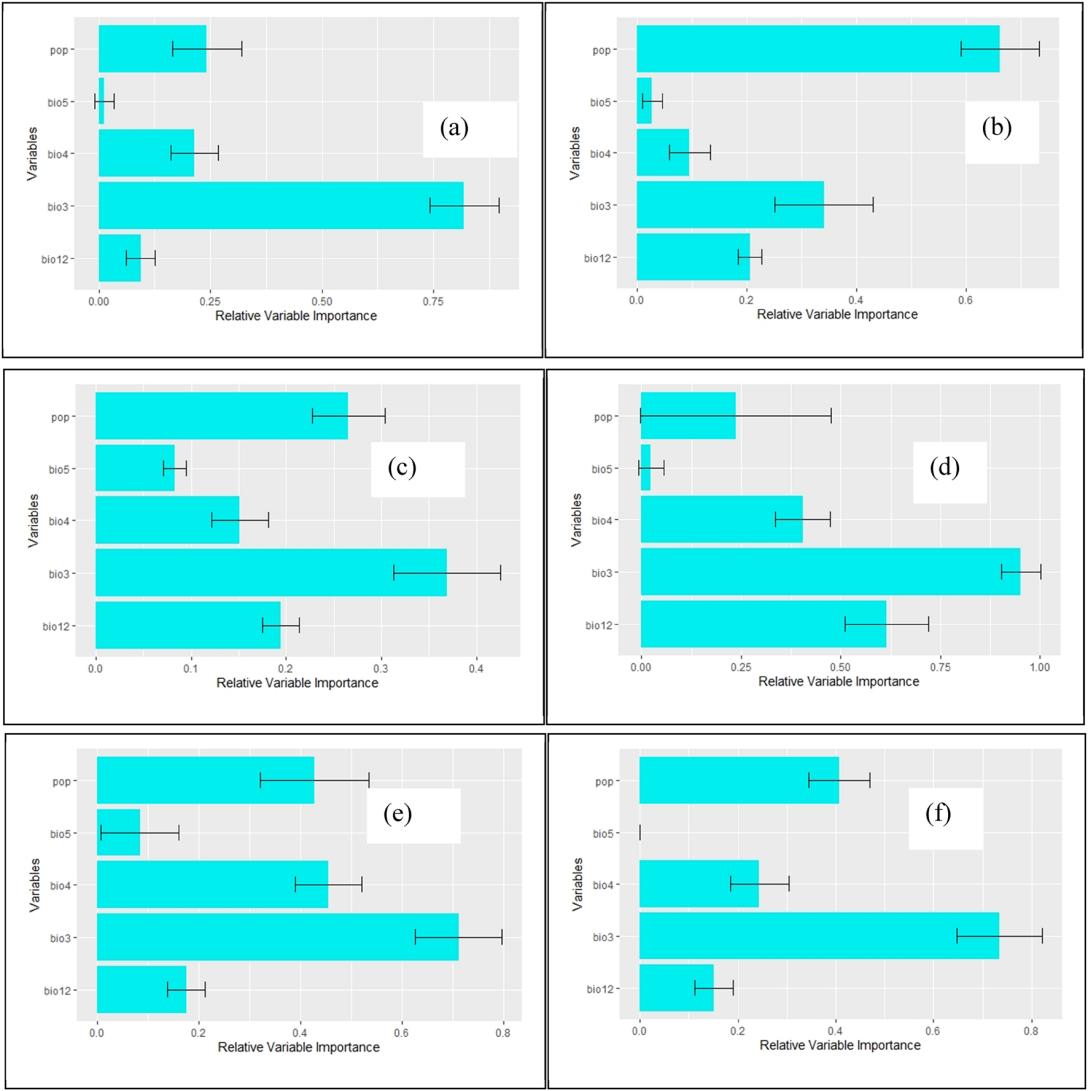
Relative importance of the environmental variables in the models: (**a**) Maxent, (**b**) BRT, (**c**) RF, (**d**) GLM, (**e**) GAM, and (**f**) MARS (the histograms were generated using R version 4.1.3 (https://www.R-project.org/)).

**Figure 3:**
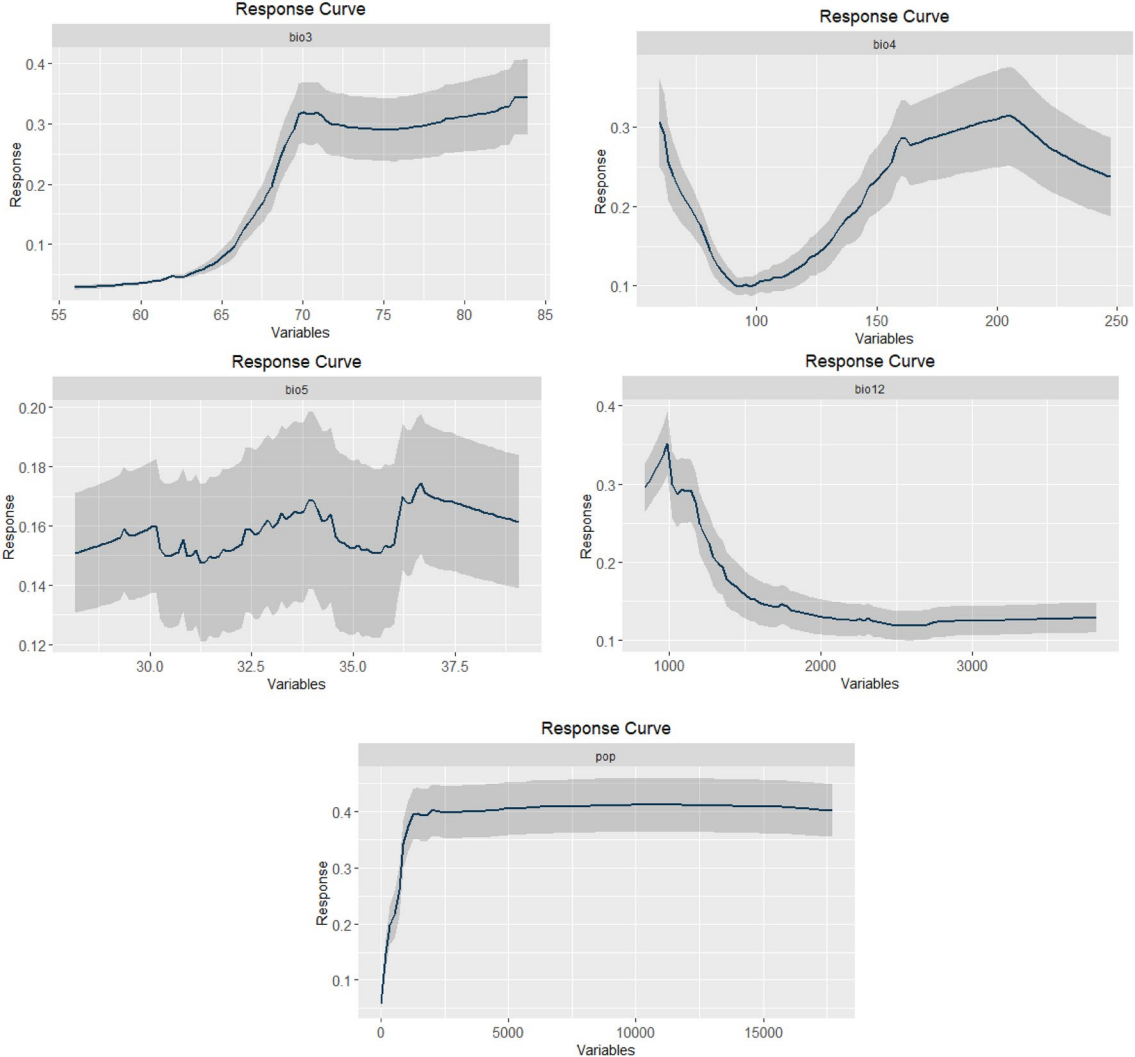
Response curves of the environmental variables retained in Machine learning models (Maxent, BRT, and RF) (the curves were generated using R version 4.1.3 (https://www.R-project.org/)).

After running the variance inflation factor (VIF) function to account for collinearity between environmental variables, followed by preliminary runs of the models to identify the most contributing variables to the models, we finally retained 5 variables in the models. They are isothermality (bio3), temperature seasonality (bio4), maximum temperature of the warmest month (bio5), annual precipitation (bio12), and population density (pop). Their relative importance is presented in Fig. 2 and the response curves of the species to the variables are presented in Fig. 3. Furthermore, the effect of climate change on the evolution of the variables retained in the models are presented in tables in Appendix 1. From Figs. 2 and 3 and Appendix 1, isothermality (bio3) is the most contributing variable, followed by population density (pop), in the two types of methods (machine learning and regression). The least contributing variable is the maximum temperature of the warmest month (bio5). According to the response curves to the variables, the suitable areas of the species will increase with the combined values of isothermality (bio3) up to 70%, annual precipitation (bio12) up to 1,000 mm, population density (pop) up to 2500 inhabitants per Km^2^, and temperature seasonality (bio4) up to 50% and 175–200%. The suitable areas of the species will globally decrease beyond those threshold values. From the results on model performance, the mean values of AUC ranged from 0.81 to 0.86, and the mean values of TSS ranged from 0.62 to 0.64 for all the models (Table 1). We therefore deduced that the models performed better than random models.

**Table 1:** Mean values of the statistics of model performance evaluation.

Methods	AUC Mean value (standard deviation)	COR Mean value (standard deviation)	TSS Mean value (standard deviation)	Deviance Mean value (standard deviation)
Maxent	0.84 (0.05)	0.48 (0.06)	0.63 (0.11)	0.74 (0.05)
BRT	0.85 (0.04)	0.57 (0.10)	0.62 (0.10)	0.46 (0.04)
RF	0.86 (0.05)	0.6 (0.06)	0.64 (0.10)	0.42 (0.06)
GLM	0.81 (0.04)	0.19 (0.07)	0.64 (0.06)	0.18 (0.01)
GAM	0.83 (0.03)	0.39 (0.05)	0.62 (0.06)	0.17 (0.02)
MARS	0.85 (0.03)	0.39 (0.04)	0.64 (0.07)	0.15 (0.01)

### Predictions at present at the scale of Africa

**Figure 4:**
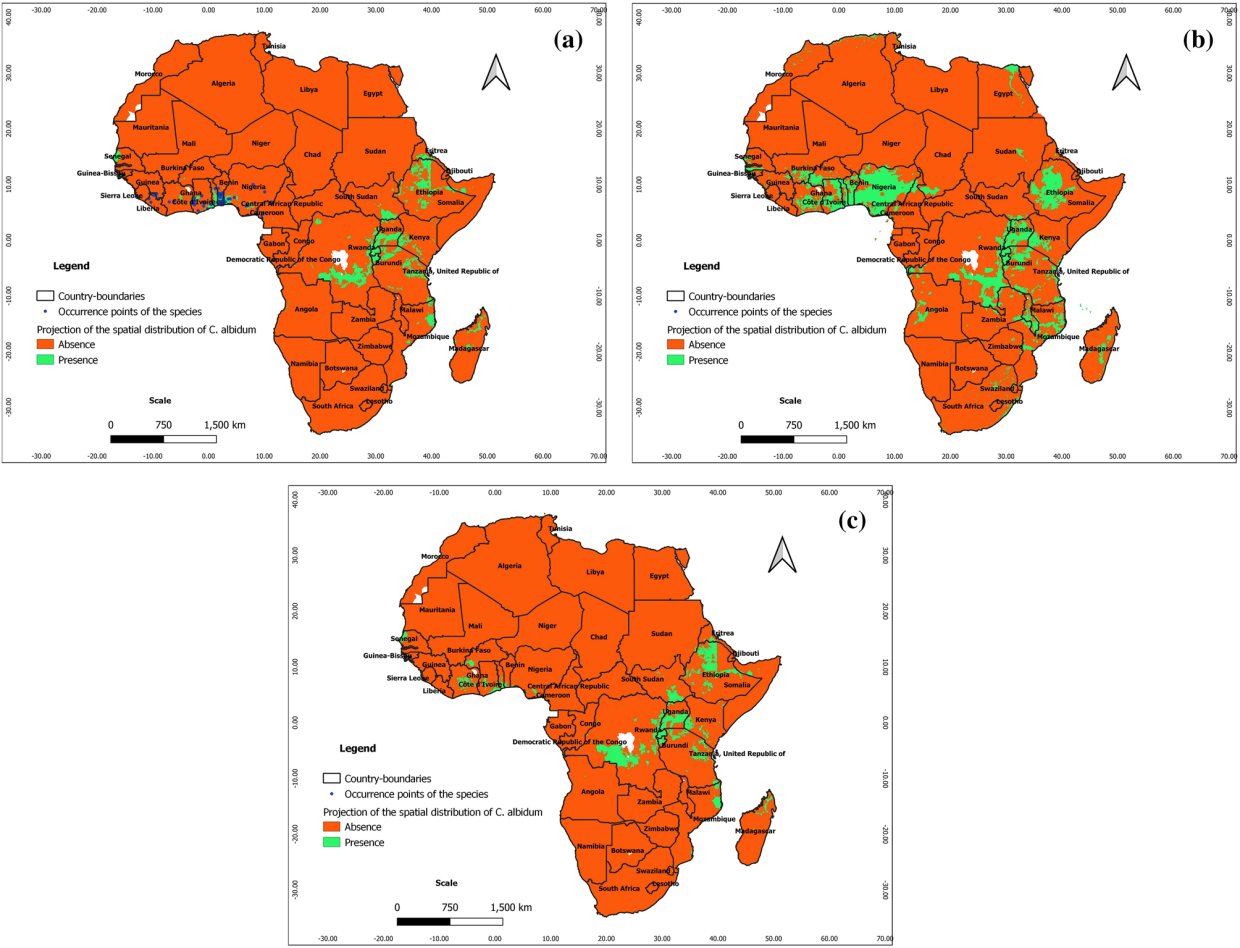
Projections of the spatial distribution of *C*. *albidum* according to Maxent across Africa: a) at present with occurrence points; b) at horizon 2055 rcp 8.5; c) at horizon 2060 SSP585 (the maps were generated using R version 4.1.3 (https://www.R-project.org/), QGIS 3.18.1 (http://qgis.osgeo.org), and WGS 84 as Coordinate Reference System).

**Figure 5:**
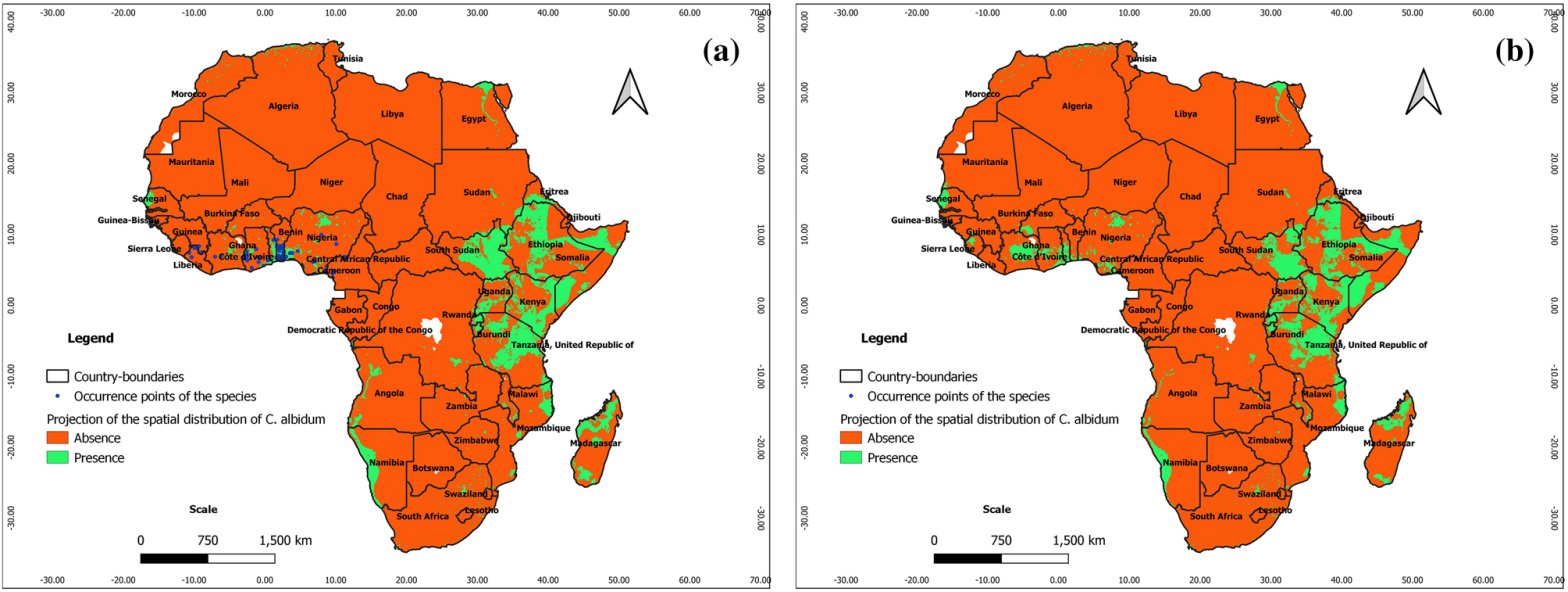
Projections of the spatial distribution of *C*. *albidum* according to BRT across Africa: a) at present with occurrence points; b) at horizon 2060 SSP585 (the maps were generated using R version 4.1.3 (https://www.R-project.org/), QGIS 3.18.1 (http://qgis.osgeo.org), and WGS 84 as Coordinate Reference System).

**Figure 6:**
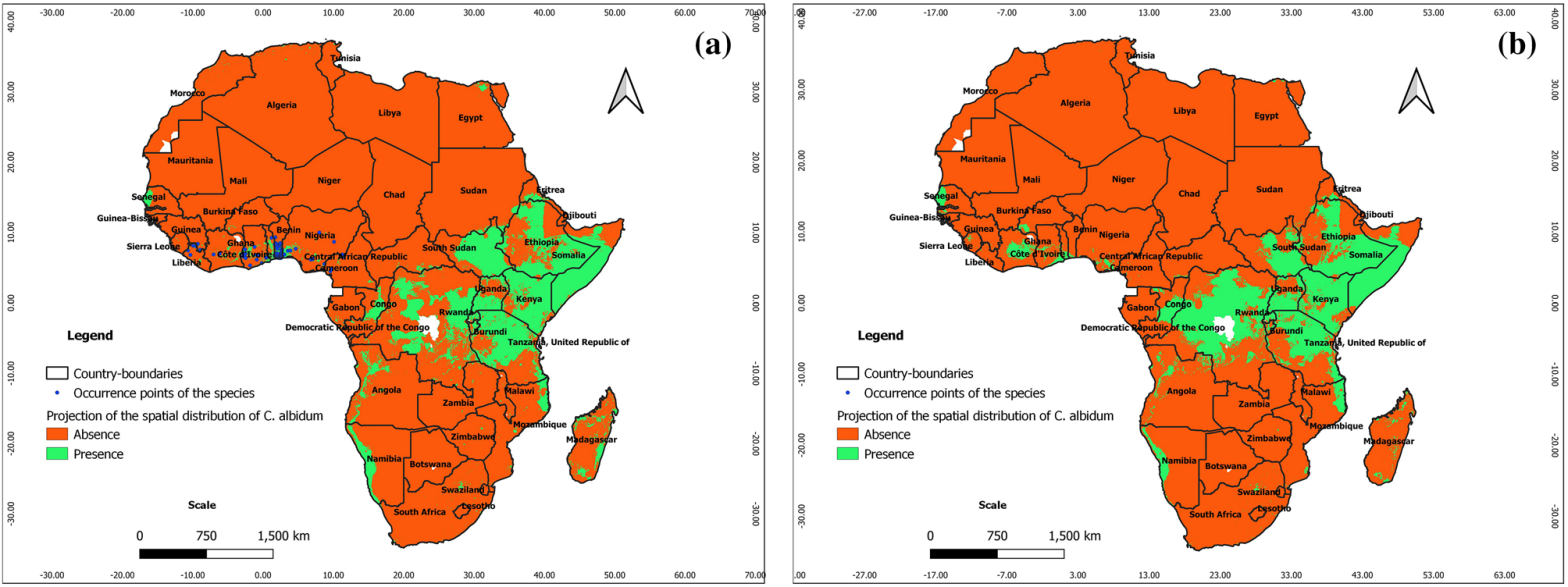
Projections of the spatial distribution of *C*. *albidum* according to RF across Africa: a) at present with occurrence points; b) at horizon 2060 SSP585 (the maps were generated using R version 4.1.3 (https://www.R-project.org/), QGIS 3.18.1 (http://qgis.osgeo.org), and WGS 84 as Coordinate Reference System).

From Figs. 4, 5, and 6, the ability of Maxent, BRT, and RF to predict real presence is noted, as their predictions at present covered most of the occurrence points of the species with additional predicted favorable areas across Africa (East and Central Africa, Center and North Madagascar). We note, however, that the predictions of favorable areas of the species with BRT and RF are more extended than those of Maxent; it is also noteworthy that under BRT and RF, the predictions of favorable areas spreading to southern and northern Africa are unrealistic with respect to the known ecology of *C. albidum*. As opposed to machine learning algorithms, the predictions of GLM, GAM, and MARS are mostly unrealistic with respect to the known ecology of *C. albidum,* a forest food tree species found in lowland rain forests of subequatorial Africa (see Electronic Supplementary Material: Appendix 2).

*NB* As lessons learned on predictions at present in Africa, Maxent, BRT, and RF are the most efficient algorithms for predicting the real presence of *C. albidum* across Africa. This is also attested by the mean values of the biserial correlation statistic (COR), which ranked Maxent, BRT, and RF higher than the other algorithms (Table 1).

From the lesson learned on the results on Africa, we only take into account the predictions of Maxent, BRT, and RF in our purposes of spatial and temporal model transfers.

### Predictions at present at the scale of Latin America

**Figure 7:**
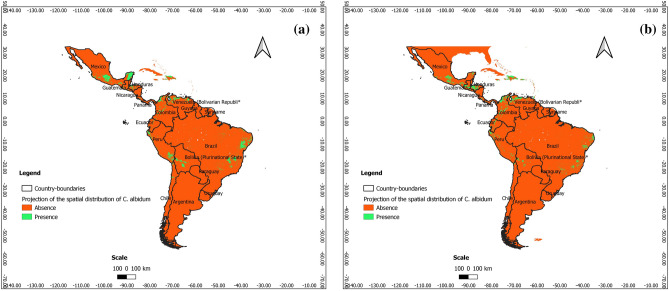
Projections of the spatial distribution of *C*. *albidum* according to Maxent across Latin America: a) at present; b) at horizon 2060 SSP585 (the maps were generated using R version 4.1.3 (https://www.R-project.org/), QGIS 3.18.1 (http://qgis.osgeo.org), and WGS 84 as Coordinate Reference System).

**Figure 8:**
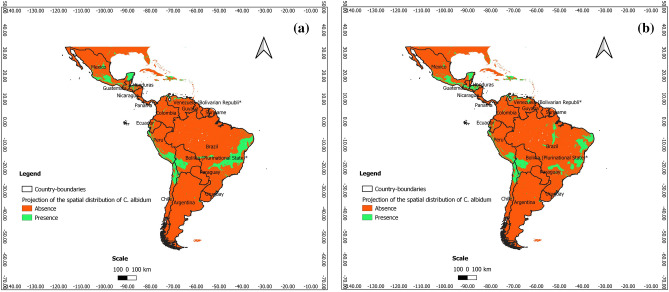
Projections of the spatial distribution of *C*. *albidum* according to BRT across Latin America: a) at present; b) at horizon 2060 SSP585 (the maps were generated using R version 4.1.3 (https://www.R-project.org/), QGIS 3.18.1 (http://qgis.osgeo.org), and WGS 84 as Coordinate Reference System).

**Figure 9:**
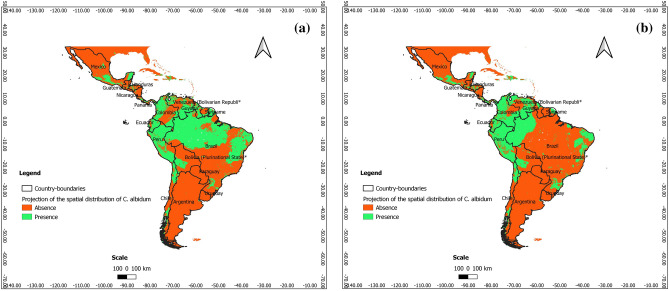
Projections of the spatial distribution of *C*. *albidum* according to RF across Latin America: a) at present; b) at horizon 2060 SSP585 (the maps were generated using R version 4.1.3 (https://www.R-project.org/), QGIS 3.18.1 (http://qgis.osgeo.org), and WGS 84 as Coordinate Reference System).

According to Figs. 7, 8 and 9, *C. albidum* can be introduced and successfully grown in parts of Latin America, especially in Central and South Mexico, Honduras, Haiti, Venezuela, Colombia, Bolivia, and East Brazil.

### Predictions at present at the scale of tropical Asia

**Figure 10:**
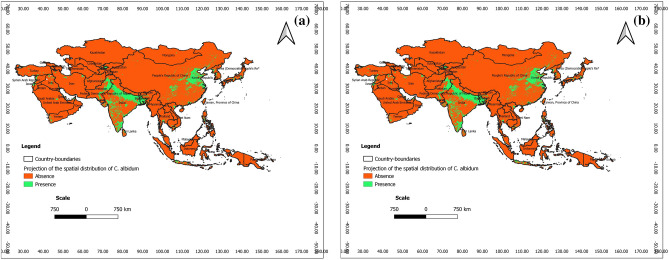
Projections of the spatial distribution of *C*. *albidum* according to BRT across Tropical Asia: a) at present; b) at horizon 2060 SSP585 (the maps were generated using R version 4.1.3 (https://www.R-project.org/), QGIS 3.18.1 (http://qgis.osgeo.org), and WGS 84 as Coordinate Reference System).

**Figure 11:**
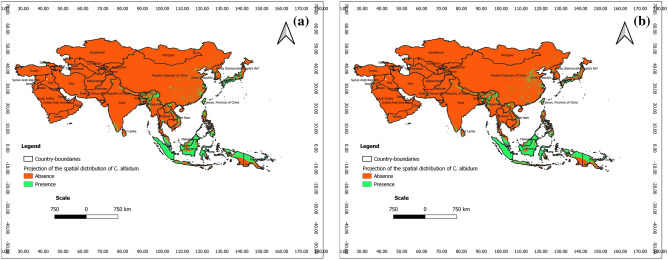
Projections of the spatial distribution of *C*. *albidum* according to RF across Tropical Asia: a) at present; b) at horizon 2060 SSP585 (the maps were generated using R version 4.1.3 (https://www.R-project.org/), QGIS 3.18.1 (http://qgis.osgeo.org), and WGS 84 as Coordinate Reference System).

According to Figs. 10 and 11, with respect to the outputs of BRT and RF, *C. albidum* can be introduced and successfully grown in parts of Asia, especially in East Pakistan, India, and Center-East China, Japan and southern parts of tropical Asia, especially Indonesia, Malaysia, and Papua New Guinea. These predictions are opposed to those of Maxent, which predicted no favorable area of *C. albidum* in Asia.

### Predictions for the future and impacts of climate change at the scale of Africa

We remind us that we used two sources of environmental climatic data, the general circulation Model for Interdisciplinary Research on Climate, Earth System version 2 for Long-term simulations (MIROCES2L) and the regional circulation model downloaded from Africlim.

#### Predictions with Africlim

According to the predictions of Maxent, under rcp 4.5 and rcp 8.5, horizon 2055, the favorable areas of *C. albidum* will extend mostly in West, East, Central, and Southern Africa as well as in East Madagascar (Fig. 4b, Appendix 3a). Compared to rcp 4.5, the predictions under rcp 8.5 sparsely showed more extensions of favorable areas in West African countries, East Africa, South DRC, and Madagascar. Under both scenarios, with respect to the present, some limited losses of favorable areas will also sparsely be noted, mostly in Center-West Togo, in some East African countries, the Center DRC, and Madagascar. As opposed to Maxent, the predictions of BRT and RF under both scenarios are unrealistic with respect to the known ecology of *C. albidum*.

#### Predictions with MIROCES2L

With respect to the predictions of Maxent**,** under scenarios SSP245 and SSP585, horizon 2060, the favorable areas of the species will be quite limited (Fig. 4c and Appendix 3b). The predictions of favorable areas of the three algorithms converged approximately and covered parts of West, East, and Central Africa as well as North Madagascar (Figs. 4c, 5b, 6b, Appendix 3). When compared to the present, the predictions of the algorithms also roughly converged and showed sparse extensions of favorable areas of the species in the same geographic subregions (Figs. 4, 5, 6, Appendix 3).

From the predictions of Maxent under both scenarios, we also noted losses of favorable areas of the species with respect to the present, sparsely in West Africa (Benin), East and Central Africa as well as across Madagascar. Additional losses of favorable areas will be noted in East Africa when the predictions of BRT and RF are considered (Figs. 4, 5, 6, Appendix 3).

The predictions of the three algorithms under SSP 585 also roughly converged when compared to the predictions under SSP245 and showed losses of favorable areas in West Africa (Benin and Togo) and across Central and East Africa as well as Madagascar (Figs. 4, 5, 6, Appendix 3).

#### Comparison of predictions under Africlim and MIROCES2L methods

**Figure 12:**
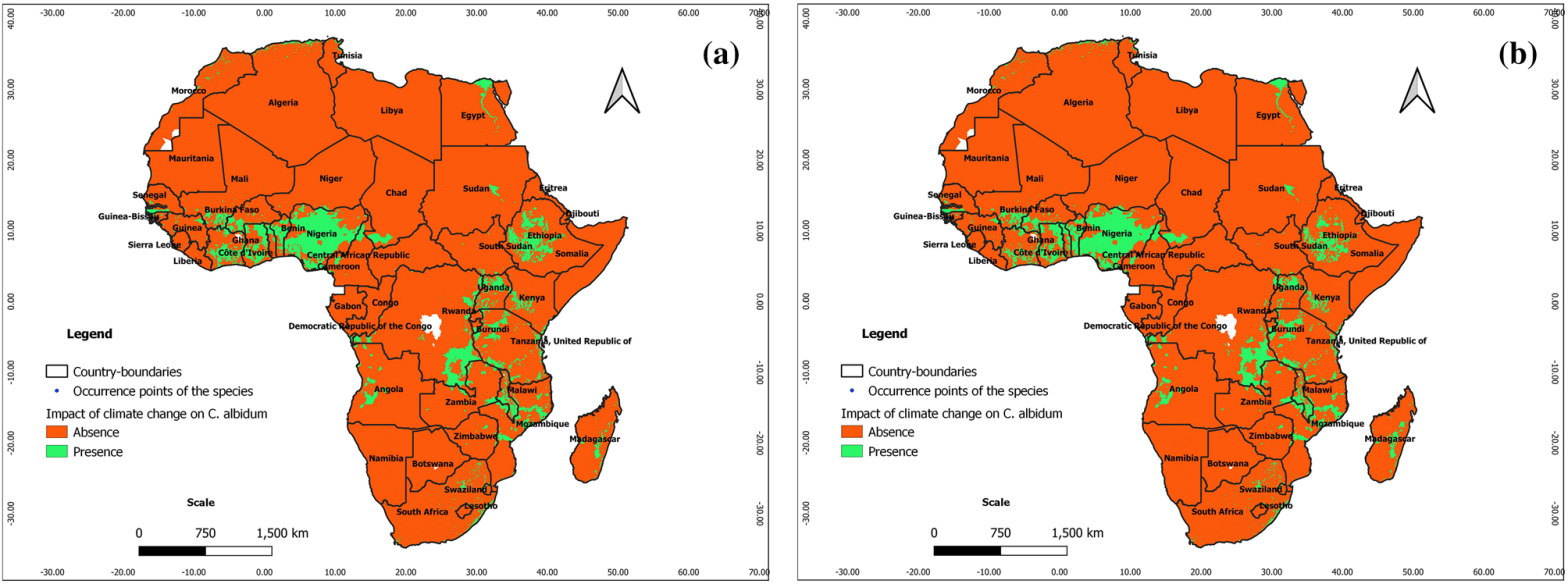
Comparison of predictions under Africlim and MIROCES2L according to Maxent: a) extension of favorable area of the species under Africlim rcp 4.5 horizon 2055 with respect to MIROCES2L SSP245 horizon 2060; b) extension of favorable area under Africlim rcp 8.5 horizon 2055 with respect to MIROCES2L SSP585 horizon 2060 (the maps were generated using R version 4.1.3 (https://www.R-project.org/), QGIS 3.18.1 (http://qgis.osgeo.org), and WGS 84 as Coordinate Reference System).

The predictions of favorable areas with Maxent are more extended under Africlim than under MIROCES2L (Fig. 12). Indeed, there is a significant extension of favorable areas of *C. albidum* in West, East, and Central Africa, as well as in Central Madagascar, under Africlim rcp 4.5 and rcp 8.5 than under the MIROES2L scenarios. Some losses are, however, sparsely noted in East and Central Africa and North Madagascar under Africlim rcp 4.5 and rcp 8.5 compared to the MIROCES2L scenarios.

### Lessons learned with respect to the predictions in Africa

At the scale of Africa, the predictions at present and in the future are consistent and coherent with Maxent either with Africlim, rcp 4.5 and 8.5 horizon 2055 or MIROCES2L SSP 245 and SSP 585 horizon 2060. The predictions with Africlim are, however, more extensive and realistic with respect to the known ecology of the species than with MIROCSE2L. For BRT and RF, the predictions are coherent and realistic at present and in the future only with MIROCES2L. The predictions in the future with BRT and RF are incoherent and unrealistic with Africlim as far as the known ecology of the species is concerned. Compatibility between climate models and algorithms is therefore suspected and needs further investigation.

### Predictions for the future and impacts of climate change at the scale of Latin America

The predictions of the three algorithms converged and showed that under MIROCES2L, scenarios SSP245 and SSP585 horizon 2060, the favorable areas of *C. albidum* cover Central America (South Mexico, Guatemala, Honduras, and Haiti) and South America (Venezuela, Colombia, Ecuador, Peru, Bolivia, and Brazil) (Figs. 7, 8, 9, Appendix 4).

Compared to the present, with Maxent and BRT, under both scenarios, extensions of favorable areas of the species will be noted in the same geographical area along with some losses. The favorable areas of the species will be more extended in parts of South America (Venezuela, Colombia, Peru, and Brazil) when RF is considered (Fig. 9, Appendix 4c), although along with some losses of favorable areas.

With respect to MIROCES2L, scenario SSP245, the predictions of the three algorithms under SSP585 converged and showed that the extension of favorable areas will sparsely be noted in Central and South America, while some losses will be sparsely deplored in the same geographical areas (Figs. 7, 8, 9, Appendix 4).

### Lessons learned with respect to the predictions in America

All the algorithms used are consistent in predicting a successful introduction of *C. albidum* in Latin America, mainly in Central America (Mexico, Guatemala, Honduras, Belize, Haiti) and North–South America (Venezuela, Colombia, Ecuador, Peru, Chile, Bolivia, and Brazil).

### Predictions for the future and impacts of climate change at the scale of tropical Asia

The projection of the spatial distribution of *C. albidum* with BRT is quite complementary to that of RF (Figs. 10, 11, Appendix 5). Indeed, with the predictions of BRT under both scenarios of MIROCES2L, the species can be introduced and successfully grown in South and East Asia (East Pakistan, India, and Center-East China) (Fig. 10, Appendix 5a)**;** compared to the present, the extension of favorable areas will be sparsely noted across the same geographic areas along with limited losses of favorable areas in India. Complementary to BRT, the predictions of RF cover India, China, Taiwan, the province of China, Japan and the southern islands of the continent (Indonesia, Malaysia, PHL, North Papua New Guinea, and Malaysia) (Fig. 11, Appendix 5b)**.** With RF and compared to the present, the extension of favorable areas will be sparsely noted across the same geographic areas. Limited losses of favorable areas will, however, be deployed across the same geographic areas. Predictions of RF and BRT are opposed to those of Maxent, which predicted no favorable area of the species in tropical Asia under the same scenarios.

### Lessons learned with respect to the predictions in tropical Asia

Predictions of the spatial distribution of *C. albidum* at present and in the future under MIROCES2L, scenarios SSP245 and SSP585, horizon 2060 were coherent and even complementary between BRT and RF and showed that the species can be successfully introduced to the central and southern countries of Asia, mainly India, China, Taiwan Province of China, Japan, Indonesia, Malaysia, PHL, and North Papua New Guinea. 

## Discussion

### Ecology of the species versus environmental factors governing its spatial distribution

The fruits of *Chrysophyllum albidum* are of nutritional, sanitary, and commercial value and highly contribute to food diversity, security and poverty alleviation^53,80,81^. The present study is the first one to determine habitat suitability of *Chrysophyllum albidum* at global scale and particularly deserves great interest for people living worldwide in the tropics. *Chrysophyllum albidum* is primarily a tropical rainforest food tree species widely distributed in West, Central, and East Africa^52–54^. Temperature, rainfall, and derived environmental factors should therefore govern the spatial distribution of the species. Global greenhouse gas emissions continue to increase in 2022 and this will exacerbate climate change so that the Global Mean Surface Temperature is estimated at 1.15 ± 0.13 °C warmer than the pre-industrial baseline (1850–1900)^6^. Furthermore, with respect to the current mean annual temperature, the predicted temperature by the Regional Circulation climatic Model Africlim^60^ and the General Circulation climatic Model MIROCES2L^61^ are consistent in predicting an increase in the mean annual temperature of more than 02 °C, at horizon 2055–2060 across the continents (Appendix 1). This will exacerbate the effect of climate change on species habitats including the ecological niche of *Chrysophyllum albidum*. Isothermality (bio3) measuring the rate of the diurnal range of temperature with respect to the annual range^82^ is the most contributing variable to the models, followed by population density (pop), annual precipitation (bio12), and temperature seasonality (bio4) (Fig. 2). The most contributing variables to the models are consistent with the known ecology of the species. According to the response curves to the variables, the suitable areas of *Chrysophyllum albidum* will increase with the combined values of isothermality (bio3) up to 70%, annual precipitation (bio12) up to 1,000 mm, population density (pop) up to 2500 inhabitants per Km^2^, and temperature seasonality (bio4) up to 30% and 175–200% (Fig. 3). The suitable areas of the species will globally decrease beyond those threshold values. The predicted values of isothermality (bio3) which is about 35% (sd = 9.60%; n = 10,000) in tropical Asia^61^ and 58.8% (sd = 12.51%; n = 98,826) in Africa^60^ is still limiting the suitable areas of the spatial distribution of *Chrysophyllum albidum* in those two regions; in Latin America however, bio3 is predicted to be close to the threshold value of 70%^61^ and will increase the suitable areas of the species in that region (Fig. 3 and Appendix 1). The predicted mean values of temperature seasonality (bio 4) in Africa is around 35% (sd = 24%; n = 98,826)^60^ and is somewhat still favorable to the suitable areas of *Chrysophyllum albidum*. In Latin America and tropical Asia however, the values of bio4 are quite above the threshold value of 200%^61^ and this will limit the spatial distribution of the species (Fig. 3 and Appendix 1). The mean annual precipitation (bio12) is predicted to vary between 650 and 670 mm (sd = 633–638 mm; n = 98,826) across Africa^60,61^; from 540 to 580 mm (sd = 608–650; n = 10,000) in tropical Asia and around 1,400 mm (sd = 853–870 mm; n = 9,992) in Latin America^61^. According to the response curve of the species to bio12, the tropics are globally favorable to the suitable areas of the species (Fig. 3 and Appendix 1). Temperature is the main driver of the other climate parameters^6^ and efforts are therefore more needed to limit at individual, national, regional, and global levels, greenhouse gas emissions so as to mitigate the rise in temperature and therefore the effect of climate change on useful species like *Chrysophyllum albidum.* When we consider the population density, and referring to the mean values of inhabitants per Km^2^ of 34 (sd = 332; n = 9,992) in Latin America; 47 (sd = 295; n = 98,826) in Africa, and 141 (sd = 698; n = 10,000) in tropical Asia (Appendix 1), across the regions^66^, all the regions are globally still favorable to the spatial distribution of the species with respect to the threshold value of 2500 inhabitants per Km^2^ of the response curve of the species (Fig. 3). A reliable grid file of the population density predicting the world population densities at horizon 2050 to 2060 did not yet exist at the time of this study so that we could not make predictions of the population density in the future. We however admit that population size is rapidly increasing^83^. Habitat fragmentation and forest degradation will therefore increase across the tropics and more impede the spatial distribution of *Chrysophyllum albidum*. As a mitigating measure, we recommend the elaboration and implementation of landscape management plans at country levels so that appropriate land use vocations can be attributed to the different compartments of the countries and enable a sustainable cultivation of *Chrysophyllum albidum* across countries and regions of the tropics. Our results are supported by those of^84^, who underlined the importance of temperature seasonality (bio4) and annual precipitation (bio12) among the environmental variables governing the distribution of *Chrysophyllum albidum* in Benin. Our results also align at least partially with those of^51^; indeed, in their studies on *Chrysophyllum albidum* in Nigeria, they listed the temperature seasonality (bio3) among the contributing variables to the distribution model of the species, although with limited importance in comparison with the minimum temperature of the coldest month (bio6). Our results also align at least partially with those of^31^ although they worked on a different species. indeed in achieving the spatial distribution of *Nepeta glomerulosa* a medicinal plant species endemic to southwestern and central Asia^31^, used environmental variables including isothermality (bio 3) and annual precipitation (bio 12); they found out that the annual precipitation (bio 12) was the most contributing variable to the model.

The values of the variables in Appendix 1 are mean values; although they are calculated from 10,000 to 100,000 observations points across the study areas, variability in the values is obvious within regions as shown by the values of the standard deviation and this might relativize our conclusion on the suitable areas of *Chrysophyllum albidum*.

### Prediction performances of the modeling methods used in the study

From our results, machine learning methods (Maxent, BRT, and RF) performed better than regression methods (GLM, GAM, and MARS). Indeed, at present in Africa, the predictions of favorable areas of *C. albidum* by the machine learning methods covered at least the occurrence points of the species, while the predictions at present from the regression methods failed in that respect. This result is also attested by the values of the performance statistics AUC, TSS, and COR. AUC and TSS are correlated^78^ and measure model performance in terms of their discrimination capacity or their ability to detect real presence from absence^8,9,13,85^. In this study, the values of AUC ranged from 0.84 to 0.86 and from 0.81 to 0.85, respectively, for machine learning methods and regression methods; the TSS values were, however, less discriminant with regard to the two types of methods and ranged from 0.62 to 0.64 for either of them. Deviance measures the deviation of the overall mean prediction values with respect to the overall presence and absence of the species^8^; it is a measurement of the goodness of fit of the models. When we observe the deviance mean values of the models, however, machine learning methods had the highest deviation mean values (0.42–0.74), whereas the lowest deviation values (0.15–0.18) were observed with regression methods (Table 1). Reliability is another measure of model predictive performance and is related to the success of a model in predicting real presence^8^. In this study, the point biserial correlation (COR)^85^ is a measurement of the model’s reliability. Its values ranged from 0.48 to 0.6 for machine learning methods against 0.19 to 0.39 for regression methods (Table 1). Our results are supported by those of^85^, who compared the predictive performance of several methods, including those used in this study, on a large range of plant and animal species. On the basis of the statistics AUC and COR values, they found that BRT and Maxent were among the methods that outperformed others, such as GLM, GAM, and MARS. In their study on three cane species in China^86^ pointed out the good predictive performance of RF^47^ in their study of model transferability applied to a range of species, including birds, butterflies, and plants of Finland, found out among several modelling methods that Maxent and BRT were among the best performing ones as opposed to RF. In contrast to our results, they noted the good transferability of GLM and GAM. Our results are also in line with^49^, who noted that although GLM and GAM can prove good in model transfers, they can also generate unrealistic predictions outside the training data of the species. The results of^25^ also support our findings; indeed, they achieved the distribution models of Carpathian endemic plant *Leucanthemum rotundifolium* (Compositae) (Central Europe) and tested many algorithms; they found out that the algorithms that performed well across different datasets include BRT and those with medium performance include Maxent and RF. According to^25^, the worst predictions were obtained with algorithms including GLM and GAM. However, our findings might be relative or case dependent. Indeed^87^, modeling numerous amphibian and reptile species of Portugal, concluded that model performance strongly depends on the geographical and environmental distributions of the species being modeled. Pearson et al.^88^, in modeling potato species of South Africa, used several modeling methods and found variability in their predictions mainly due to the input type of data (presence/absence vs. presence only data) and extrapolation assumptions of the methods. They did not identify the best method for predictions. GLM, GAM, and MARS were initially conceived to use presence/absence data^88^, and since we used only presence data in our study, background point generation in modeling approaches such as the SDM package^69^ instead of real absence might limit the ability of predictions of the regression methods.

### Strategies for the conservation of *C. albidum*, a potential pan tropical forest food species, under climate and global changes

Predictive models are vital to inform decisions on natural resource management in the context of climate and global changes^49^. To appraise the impacts of climate change on the management of the population of *C. albidum* in the tropics, we performed temporal and spatial transfers of the models achieved. Model transfers have many advantages and can guide decision making in resource management and biodiversity conservation^48–50^. The spatial model transfers at present across Africa with the outputs of Maxent, BRT, and RF were quite consistent with the known ecology of *Chrysophyllum albidum*. We can therefore take the prediction of the species at present in Madagascar as realistic and then introduce and cultivate the species in that country.

When we consider environmental data from Africlim^60^, the predictions of the models developed with Maxent showed a positive impact of climate change on the spatial distribution of the species across Africa. The species is therefore not at risk in Africa with respect to climate change at least within the threshold values of the most contributing variables to the models. Our results are supported by those of^51^ and^84^, who found out under climate change, an extension of the favorable areas of the species in Nigeria and in Benin, respectively.

Globally, spatial and temporal model transfers with the outputs of Maxent are consistent and coherent with the known ecology of the species either with Africlim under the RCP4.5 and RCP8.5 horizons 2055 or MIROCES2L SSP245 and SSP585 horizons 2060. The predictions with Africlim were, however, more extensive and realistic than those with MIROCES2L. For BRT and RF, the predictions were coherent and realistic at present and in the future only with MIROCES2L. Indeed, with respect to the known ecology of the species, the predictions in the future with BRT and RF are incoherent and unrealistic with Africlim. Environmental data from Africlim are derived from regional circulation models downscaled to fit the realities of Africa made of a high density of populations in coastal areas and marked mountainous topography^60^. The outputs with Africlim should therefore be more realistic than those from general circulation models like MIROCES2L^61^. A problem of compatibility between algorithms and the source of environmental data can therefore be suspected and needs further investigation.

Spatial transfers can contribute to species relocations or reintroductions; promotion of species-habitat conservation and forecasting areas vulnerable to invasion^46–50^. Indeed, spatial transfers are quite exciting when they can help introduce new species in potentially favorable areas. In this study, we transferred the models to Latin America and tropical Asia. All the algorithms used were consistent in predicting a successful introduction of *C. albidum* in Latin America both at present and in the future, mainly in Central America (Mexico, Guatemala, Honduras, Belize, and Haiti) and North South America (Venezuela, Colombia, Ecuador, Peru, Chile, Bolivia, and Brazil). Predictions of the spatial distribution of *C. albidum* at present and in the future under SSP245 and SSP585 horizon 2060 were coherent and even complementary between BRT and RF and showed that the species can be successfully introduced to the central and southern countries of Asia, mainly India, China, Taiwan Province of China, Japan, Indonesia, Malaysia, PHL, and North Papua New Guinea.

The species is native to Africa; in the predicted suitable areas of the continent, we recommend introducing the species where it is absent and increasing its stocks where it is already present but at low densities. In Latin America and tropical Asia, we recommend the introduction and cultivation of the species in the predicted suitable areas with a follow-up care to enable its establishment, along with vegetation inventories in order to discover, likely, sister or vicarious species of *Chrysophyllum albidum* that can be new to Science.

At the regional levels (Africa, Latin America, and tropical Asia), the variable soil was not considered in the modeling process. At the local scale, however, where the introduction of the species is envisioned, types of soil can become a limiting factor for the successful establishment of the species^89^. Indeed, *C. albidum* is mostly found on ferrallitic well-drained soils in its natural range; its introduction elsewhere should therefore take into account the appropriate types of soils to enable success.

## Conclusion

*Chrysophyllum albidum* is a forest food tree species of the Sapotaceae family bearing large berries of nutrition, sanitary, and commercial value in many African countries. The climatic models used in the study globally predict an increase of the suitable areas of the species across continents. However the response curves of the species to the contributing variables to its spatial distribution clearly show that the suitability of its areas is subjected to threshold values of the most contributing variables beyond which its suitable areas will decrease. With respect to human pressure represented in the models by population density (pop), in the context of a growing human population, we recommend the elaboration and implementation of landscape management plans at country levels so that appropriate land use vocations can be attributed to the different compartments of the countries and enable a sustainable cultivation of *Chrysophyllum albidum* across countries and regions of the tropics. Apart from its natural range in tropical Africa, *Chrysophyllum albidum* can be successfully introduced and grown worldwide in the tropics, especially in Latin America and tropical Asia. We therefore recommend the introduction and cultivation of the species in the predicted suitable areas of Latin America and tropical Asia, along with vegetation inventories in order to discover likely, sister or vicarious species of *Chrysophyllum albidum* that can be new to Science. The regional circulation model Africlim is more efficient than the general circulation model MIROCES2L in predicting realistic suitable areas of the species in Africa. We therefore recommend to the authors of Africlim an update of Africlim models to comply with the sixth Assessment Report (AR6) of IPCC.

## Supplementary Information

Supplementary Information 1.

Supplementary Information 2.

Supplementary Information 3.
