# Supplementary material for: Ecological niche model transferability of the white star apple (Chrysophyllum albidum G. Don) in the context of climate and global changes
Source: Sci Rep. 2023 Feb 10;13:2430. doi: 10.1038/s41598-023-29048-3 (PMC9918511; doi:10.1038/s41598-023-29048-3)
Supplement: Supplementary file 1 — Supplementary Information 1. [file 41598_2023_29048_MOESM1_ESM.zip › GANGLO_Appendices/Ganglo_Appendices_captions_SR.docx]

**Appendix 1a**: Predictions with respect to Africlim (Platts et al. 2015) at Africa level of the annual mean temperature (bio1) and the variables retained in the models (bio3, bio4, bio5, bio12, pop)

| Variables | Mean value at present (standard deviation) | Predictions of Africlim horizon 2055 (n=98,826) | |
| --- | --- | --- | --- |
|  |  | Mean value RCP4.5  (standard deviation) | Mean value RCP8.5  (standard deviation) |
| Annual meam temperatre (bio1 °C) | 23° 53' 24"  (3° 32') | 26° 3' 36"  (3° 42') | 26° 49' 48"  (3° 44') |
| Isothermality (bio3, %) | 58.80  (12.51) | 58.63  (123.46) | 58.36  () |
| Temperature Seasonality (bio4, %) | 360.01  (245.03) | 35.30  (23.97) | 35.56  (24.17) |
| Max Temperature of Warmest Month (bio5, °C) | 35° 30'  (4° 55') | 38° 10' 48"  (51° 43') | 38° 58' 12"  (52° 20') |
| Annual Precipitation (bio12, mm) | 647.21  (622.91) | 656.83  (633.43) | 661.63  (637.26) |
| Population density per Km² (pop, CIESIN 2018) | 46.52  (295.39) | - | - |

**Appendix 1b**: Predictions with respect to MIROCES2L (Hajima et al. 2020) at Africa level of the annual mean temperature (bio1) and the variables retained in the models (bio3, bio4, bio5, bio12, pop)

| Variables | Mean value at present  (standard deviation) | Predictions of MIROCES2L horizon 2060 (n=98,826) | |
| --- | --- | --- | --- |
|  |  | Mean value  ss245  (standard deviation) | Mean value  ss585  (standard deviation) |
| Annual meam temperatre (bio1 °C) | 23° 53' 24"  (3° 32') | 25° 38'  (3° 33') | 26° 11'  (3° 35') |
| Isothermality (bio3, %) | 58.80  (12.51) | 57.76  (12.83) | 57.18  (12.82) |
| Temperature Seasonality (bio4, %) | 360.01  (245.03) | 369.07  (253.29) | 376.85  (253.50) |
| Max Temperature of Warmest Month (bio5, °C) | 35° 30'  (4° 55') | 37° 31'  (5° 5') | 38° 28'  (5° 4') |
| Annual Precipitation (bio12, mm) | 647.21  (622.91) | 671.99  (644.39) | 682.17  (653.41) |
| Population density per Km² (pop, CIESIN 2018) | 46.52  (295.39) | - | - |

**Appendix 1c**: Predictions with respect to MIROCES2L (Hajima et al. 2020) at Latin America level of the annual mean temperature (bio1) and the variables retained in the models (bio3, bio4, bio5, bio12, pop)

| Variables | Mean value at present  (standard deviation) | Predictions of MIROCES2L horizon 2060 (n=9,992) | |
| --- | --- | --- | --- |
|  |  | Mean value  ss245  (standard deviation) | Mean value  ss585  (standard deviation) |
| Annual mean temperature (bio1 °C) | 21° 16'  (6° 10') | 22° 53'  (6° 25') | 23° 12'  (6° 23') |
| Isothermality (bio3, %) | 67.49  (12.82) | 65.77  (12.69) | 66.04  (12.99) |
| Temperature Seasonality (bio4, %) | 213.93  (184.37) | 228.45  (182.69) | 231.02  (188.44) |
| Max Temperature of Warmest Month (bio5, °C) | 30° 17'  (5° 4') | 32° 35'  (5° 26') | 32° 47'  (5° 29') |
| Annual Precipitation (bio12, mm) | 1434.96  (869.12) | 1399.66  (853.50) | 1413.50  (864.61) |
| Population density per Km² (pop, CIESIN 2018) | 34.53  (331.91) | - | - |

**Appendix 1d**: Predictions with respect to MIROCES2L (Hajima et al. 2020) at tropical Asia level of the annual mean temperature (bio1) and the variables retained in the models (bio3, bio4, bio5, bio12, pop)

| Variables | Mean value at present  (standard deviation) | Predictions of MIROCES2L horizon 2060 (n=10,000) | |
| --- | --- | --- | --- |
| - | - | Mean value  ss245  (standard deviation) | Mean value  ss585  (standard deviation) |
| Annual meam temperatre (bio1 °C) | 12° 25'  (10° 31') | 14° 42'  (10° 5') | 15° 21'  (10° 1') |
| Isothermality (bio3, %) | 35.00  (9.60) | 34.98  (9.14) | 34.89  (9.14) |
| Temperature Seasonality (bio4, %) | 926.39  (377.95) | 924.05  (385.67) | 925.25  (380.11) |
| Max Temperature of Warmest Month (bio5, °C) | 30° 40'  (8° 30') | 33° 2'  (8° 29') | 33° 42'  (8° 32') |
| Annual Precipitation (bio12, mm) | 544.19  (608.65) | 569.45  (628.72) | 577.50  (646.47) |
| Population density per Km² (pop, CIESIN 2018) | 141.54  (698.27) | - | - |


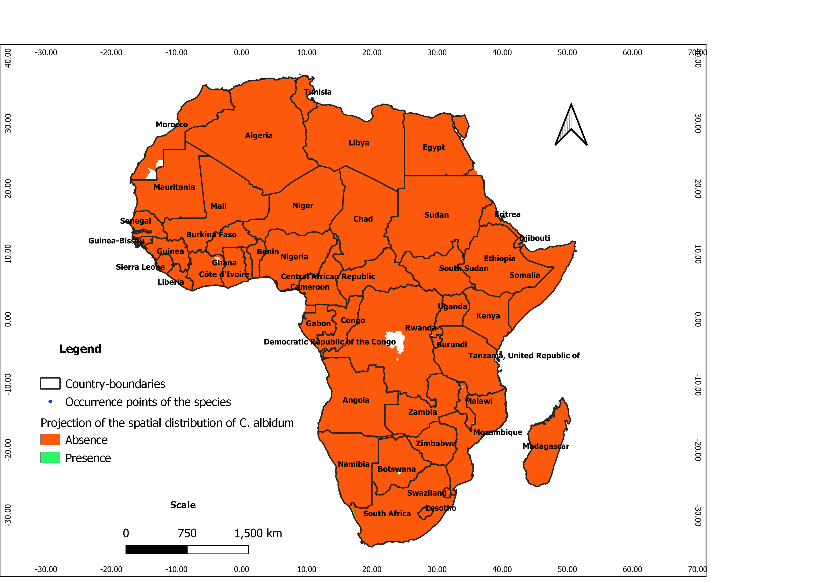

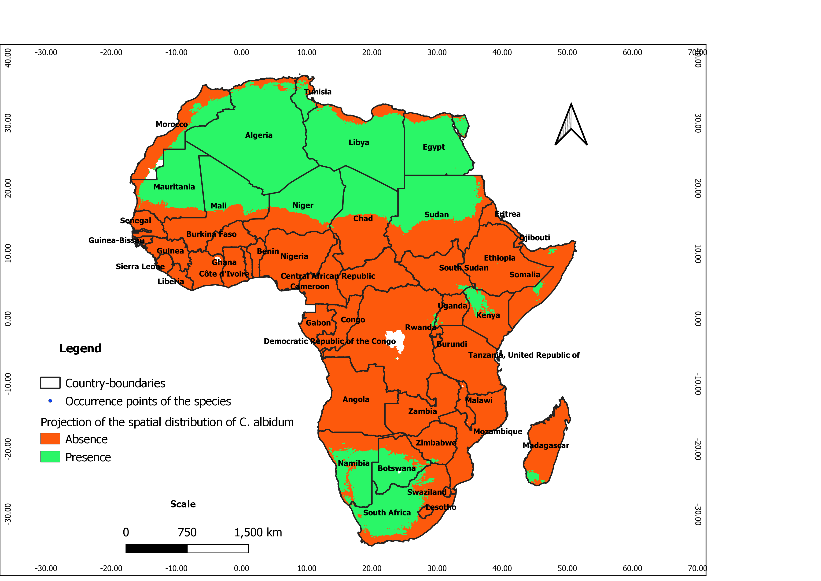


(b)

(a)

**Appendix 2**: Projection at present of the spatial distribution of C. *albidum* across Africa: a) according to GLM; b) according to GAM or MARS (the maps were generated using R version 4.1.3 (<https://www.R-project.org/>), QGIS 3.18.1 ([http://qgis.osgeo.org](http://qgis.osgeo.org/)), and WGS 84 as Coordinate Reference System).

(a)

(b)

(d)


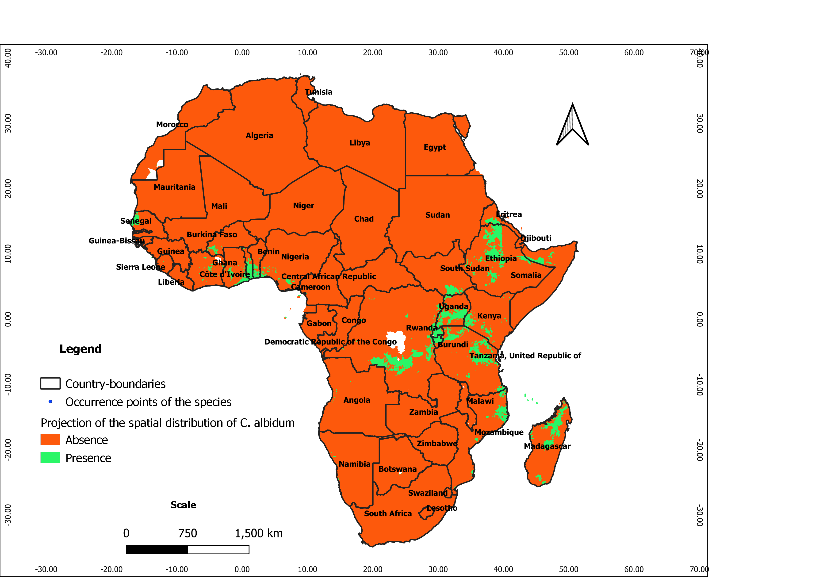

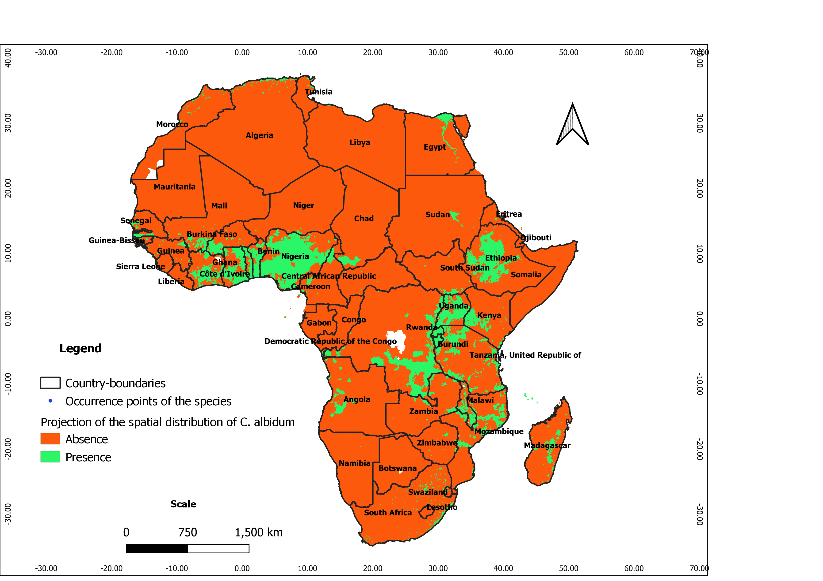


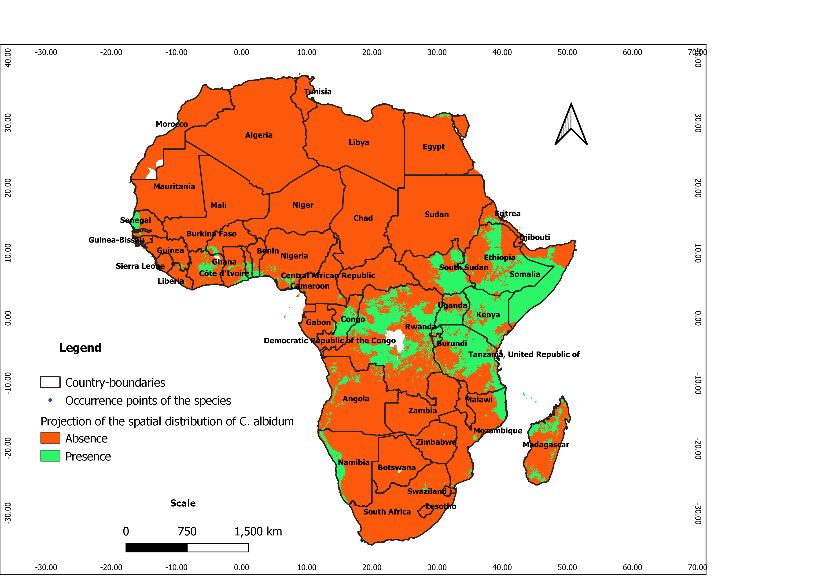

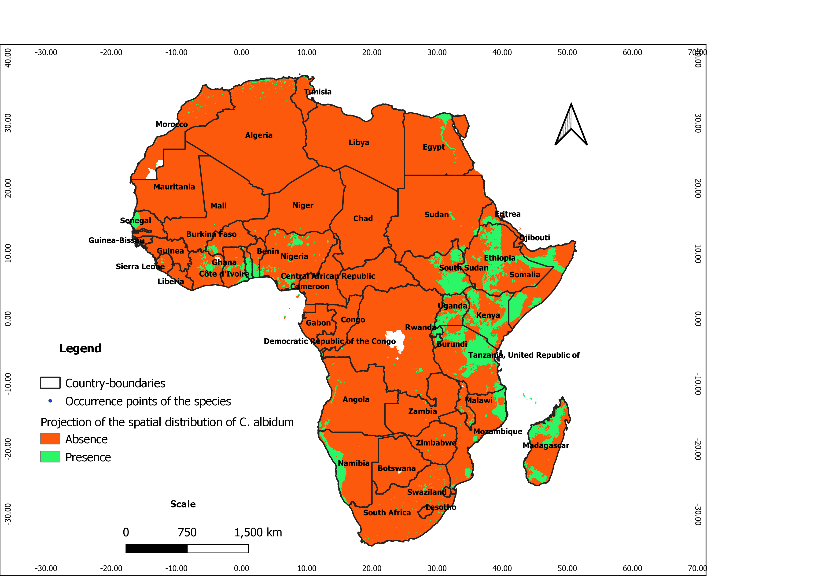


(c)

**Appendix 3**: Projection of the spatial distribution of C. *albidum* across Africa: a) at horizon 2055, rcp 4.5 according to Maxent; b) at horizon 2060 SSP245 according to Maxent; c) at horizon 2060 SSP245 according to BRT ; d) at horizon 2060 SSP245 according to RF (the maps were generated using R version 4.1.3 (<https://www.R-project.org/>), QGIS 3.18.1 ([http://qgis.osgeo.org](http://qgis.osgeo.org/)), and WGS 84 as Coordinate Reference System).


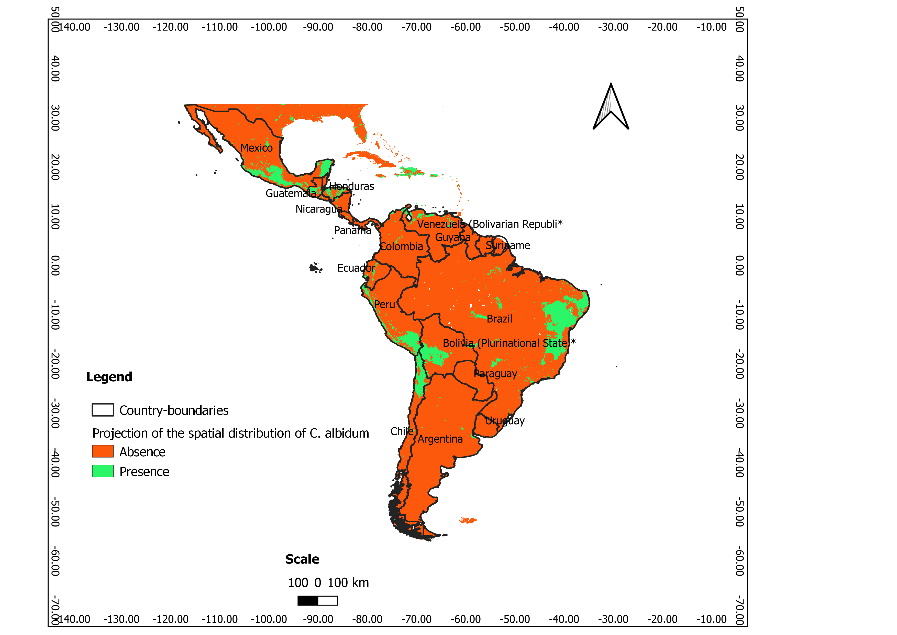

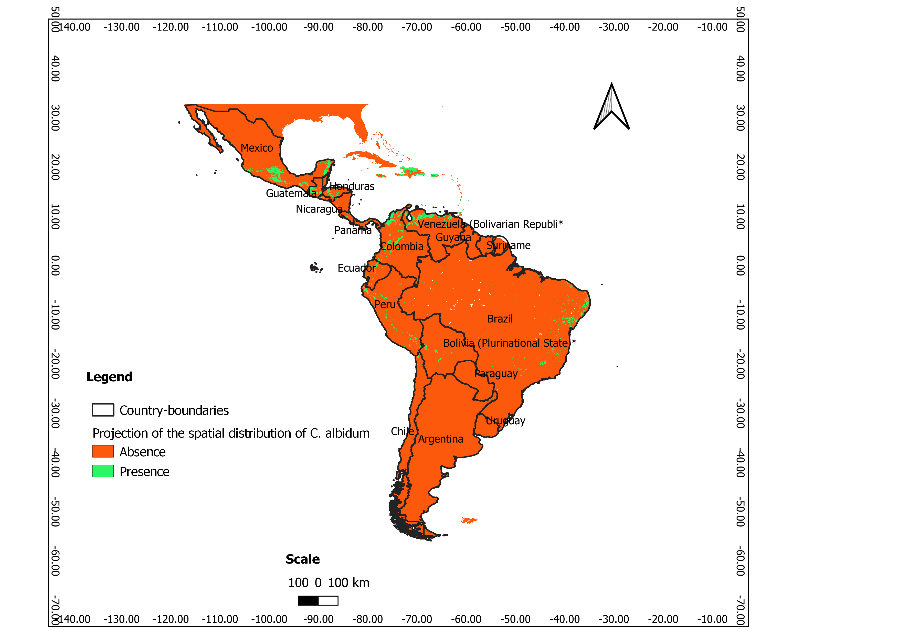


(b)

(a)

(c)


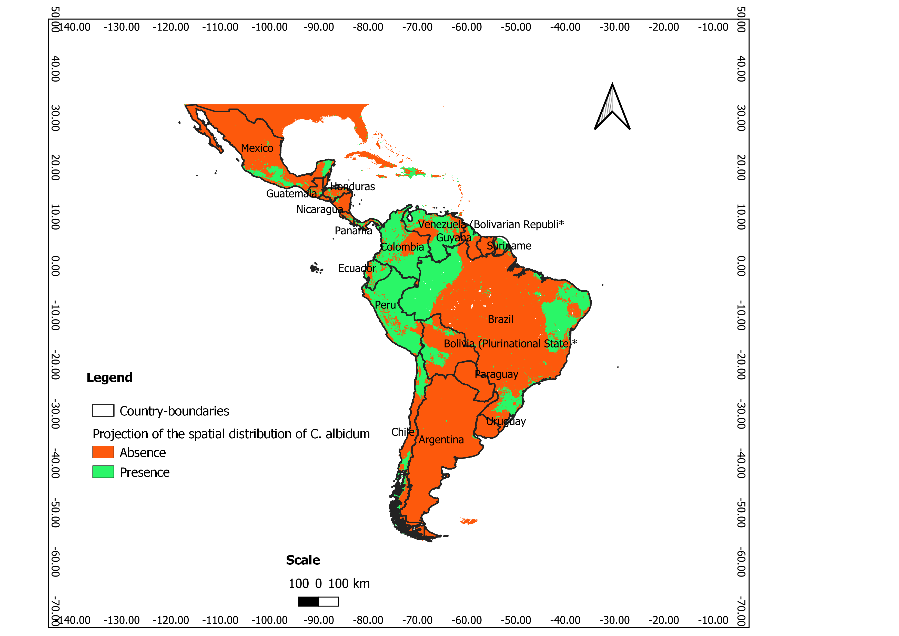


**Appendix 4**: Projection of the spatial distribution of C. *albidum* across Latin America: a) at horizon 2060 SSP245 according to Maxent; b) at horizon 2060 SSP245 according to BRT; c) at horizon 2060 SSP245 according to RF (the maps were generated using R version 4.1.3 (<https://www.R-project.org/>), QGIS 3.18.1 ([http://qgis.osgeo.org](http://qgis.osgeo.org/)), and WGS 84 as Coordinate Reference System).


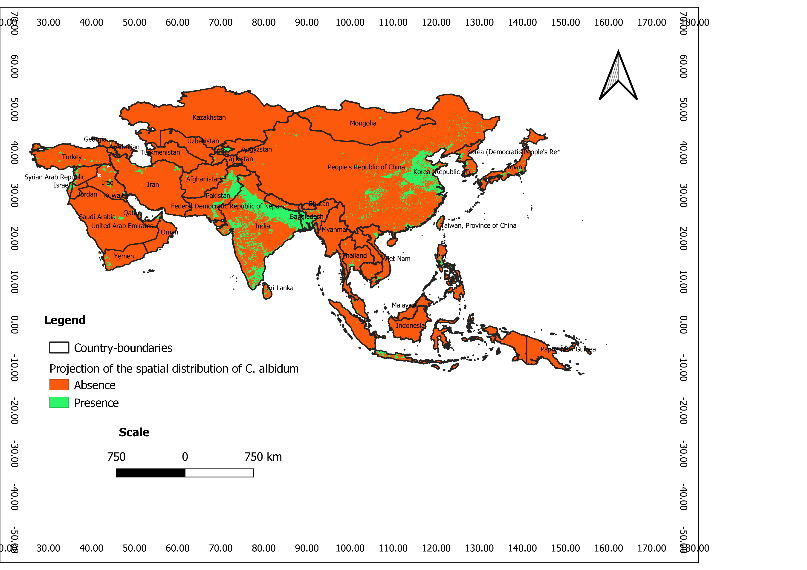


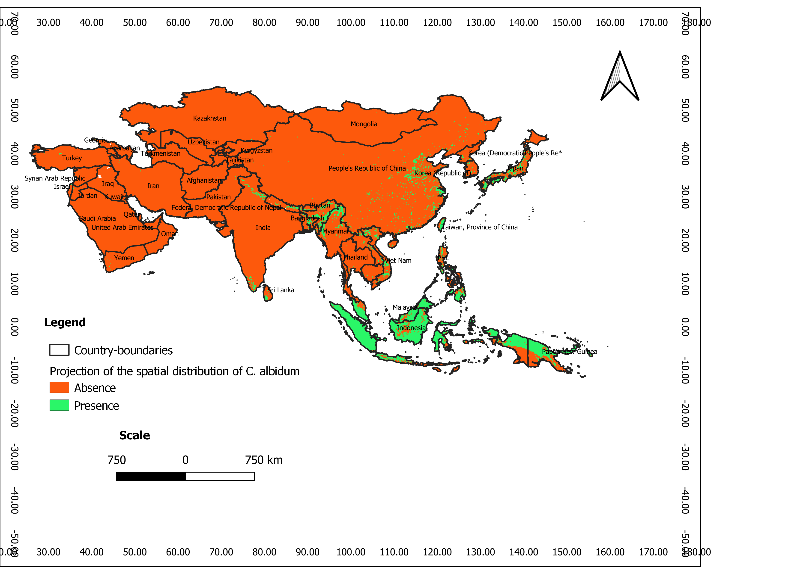


(b)

(a)

**Appendix 5**: Projection of the spatial distribution of C. *albidum* across Tropical Asia: a) at horizon 2060 SSP245 according to BRT; b) at horizon 2060 SSP245 according to RF (the maps were generated using R version 4.1.3 (<https://www.R-project.org/>), QGIS 3.18.1 ([http://qgis.osgeo.org](http://qgis.osgeo.org/)), and WGS 84 as Coordinate Reference System).
