# Supplementary figures and images for: Ecological niche model transferability of the white star apple (Chrysophyllum albidum G. Don) in the context of climate and global changes
Source: Sci Rep. 2023 Feb 10;13:2430. doi: 10.1038/s41598-023-29048-3 (PMC9918511; doi:10.1038/s41598-023-29048-3)

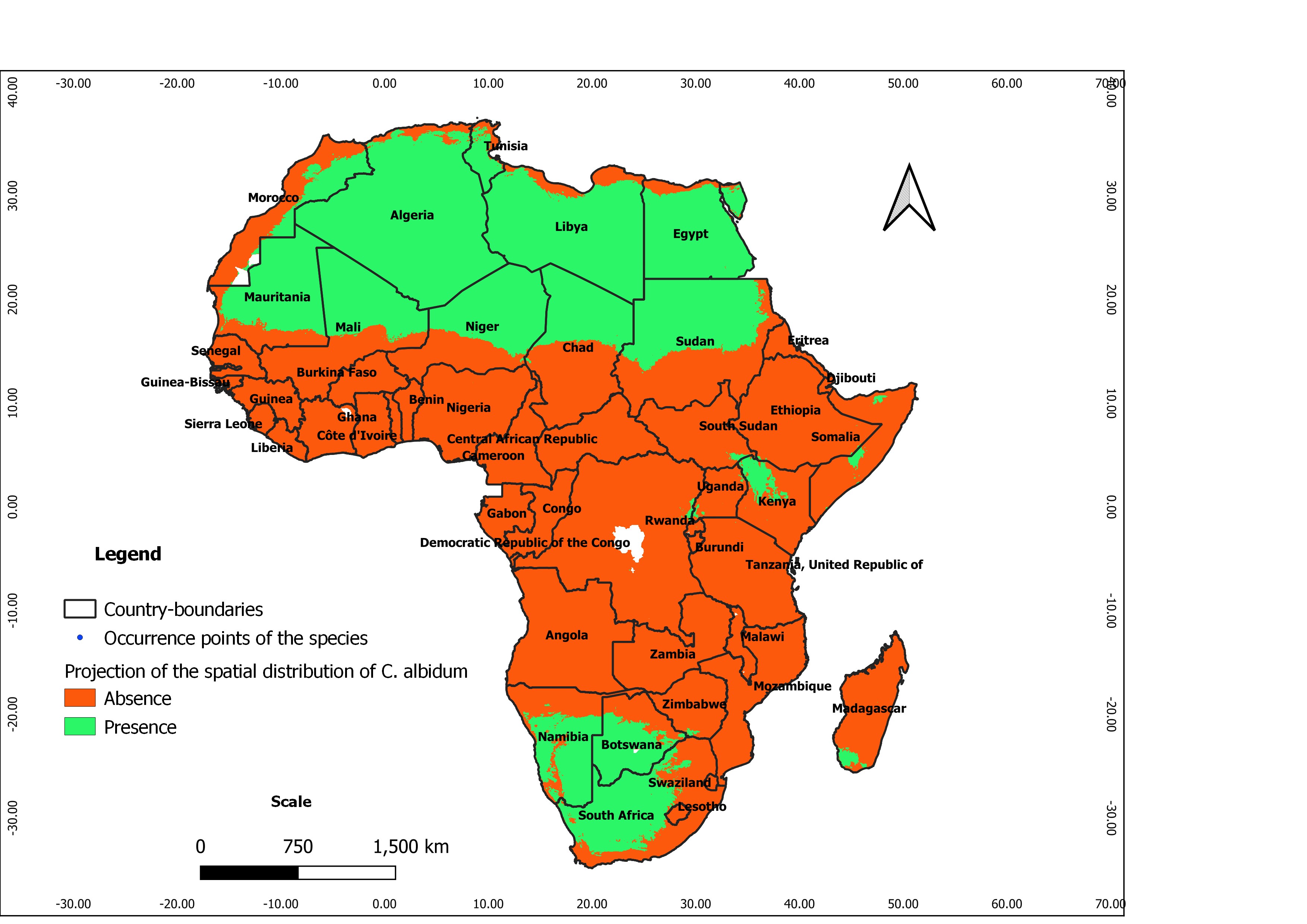

Supplement: Supplementary file 1 — Supplementary Information 1. [file 41598_2023_29048_MOESM1_ESM.zip › GANGLO_Appendices/Appendix_2/Appendix_2a.jpg]

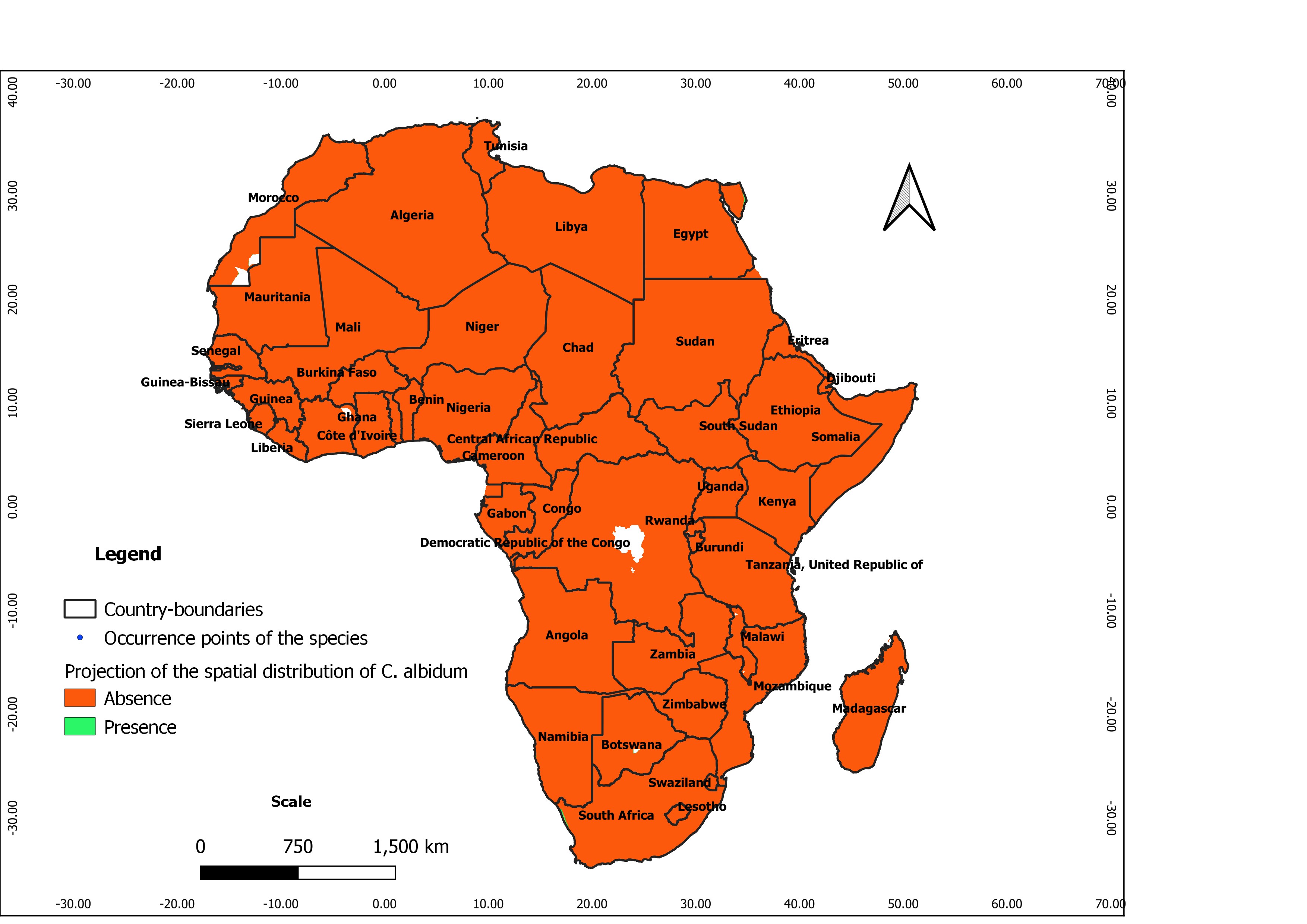

Supplement: Supplementary file 1 — Supplementary Information 1. [file 41598_2023_29048_MOESM1_ESM.zip › GANGLO_Appendices/Appendix_2/Appendix_2b.jpg]

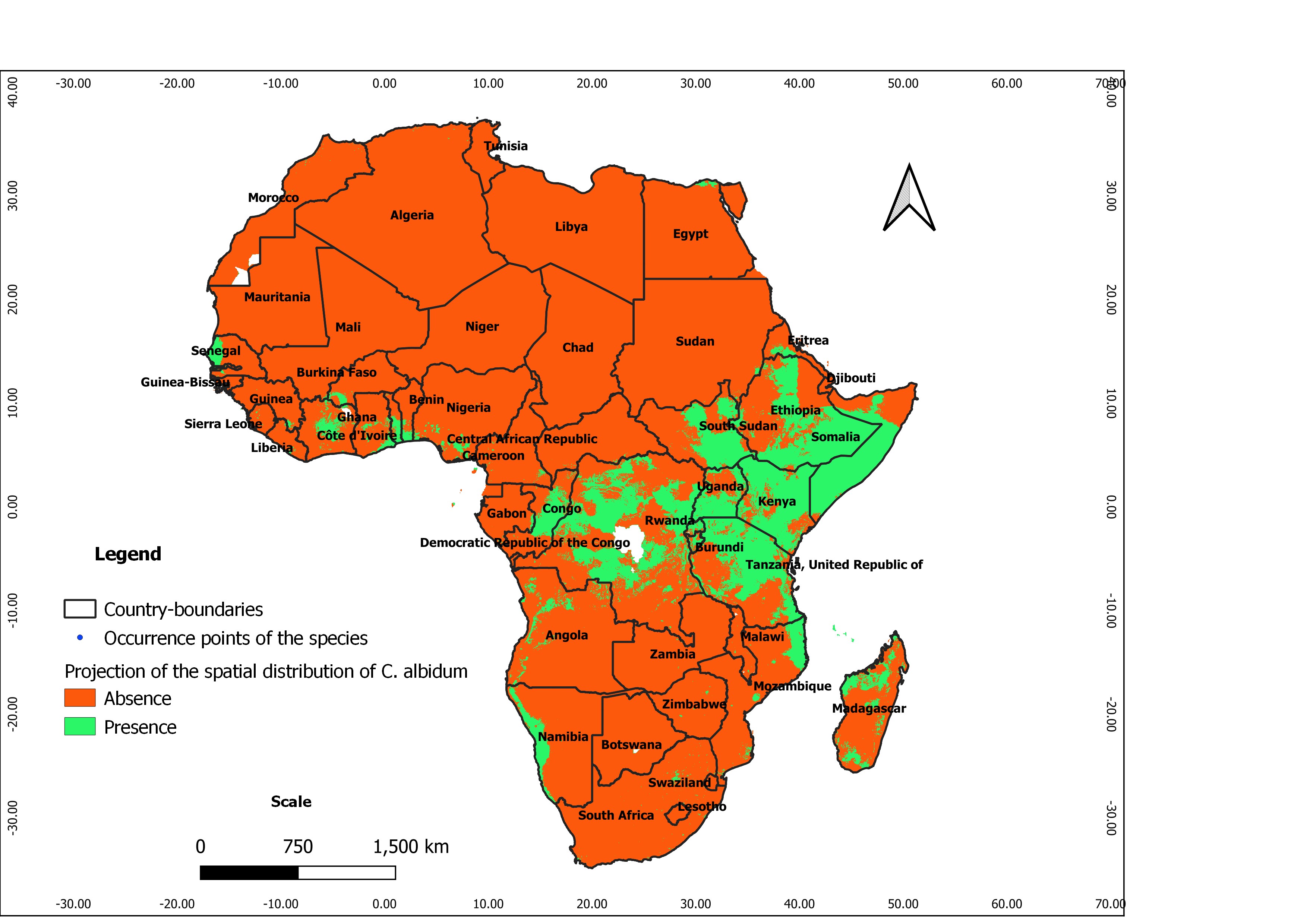

Supplement: Supplementary file 1 — Supplementary Information 1. [file 41598_2023_29048_MOESM1_ESM.zip › GANGLO_Appendices/Appendix_3/Appendix_3d.jpg]

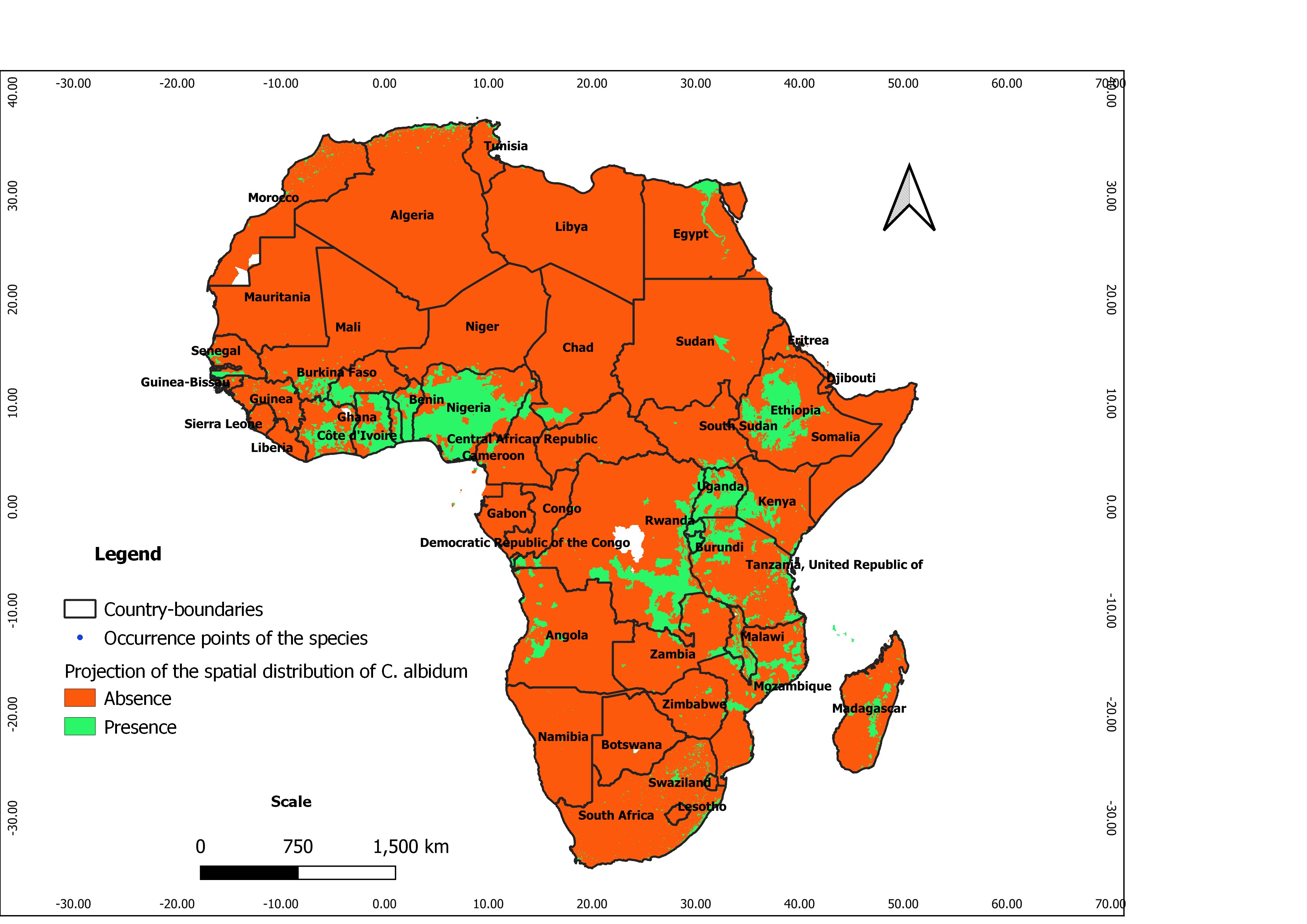

Supplement: Supplementary file 1 — Supplementary Information 1. [file 41598_2023_29048_MOESM1_ESM.zip › GANGLO_Appendices/Appendix_3/Appendix_3a.jpg]

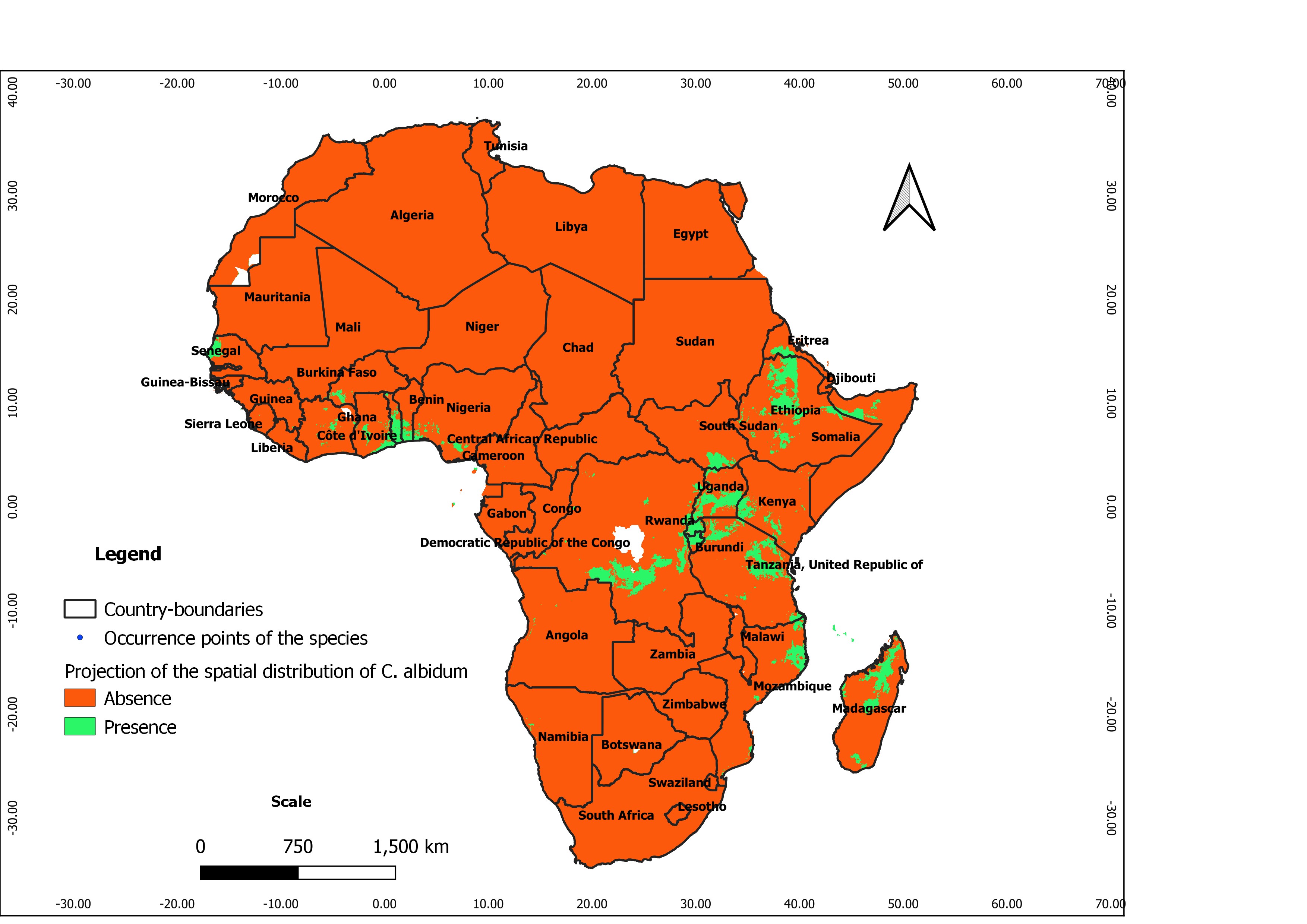

Supplement: Supplementary file 1 — Supplementary Information 1. [file 41598_2023_29048_MOESM1_ESM.zip › GANGLO_Appendices/Appendix_3/Appendix_3b.jpg]

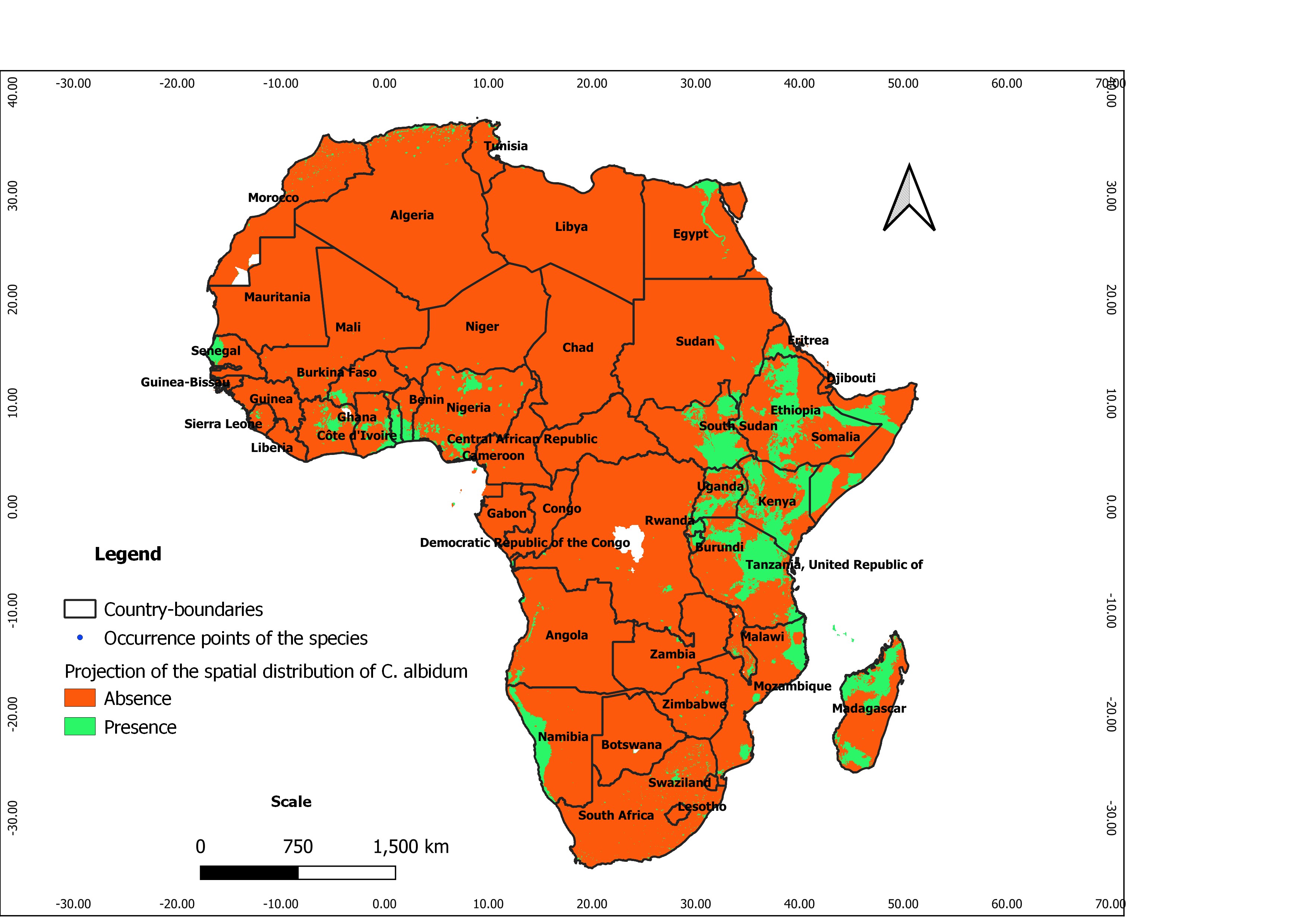

Supplement: Supplementary file 1 — Supplementary Information 1. [file 41598_2023_29048_MOESM1_ESM.zip › GANGLO_Appendices/Appendix_3/Appendix_3c.jpg]

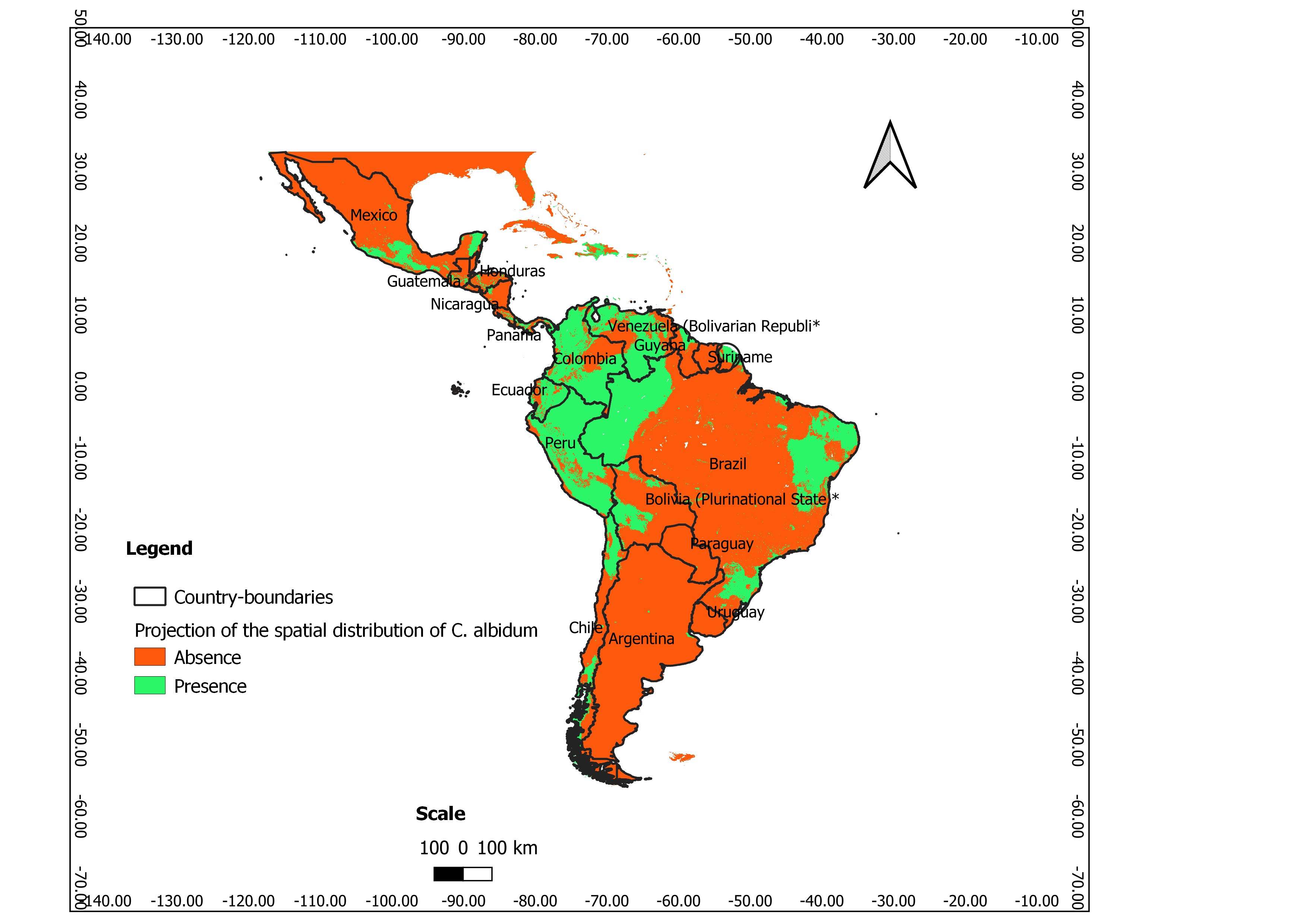

Supplement: Supplementary file 1 — Supplementary Information 1. [file 41598_2023_29048_MOESM1_ESM.zip › GANGLO_Appendices/Appendix_4/Appendix_4c.jpg]

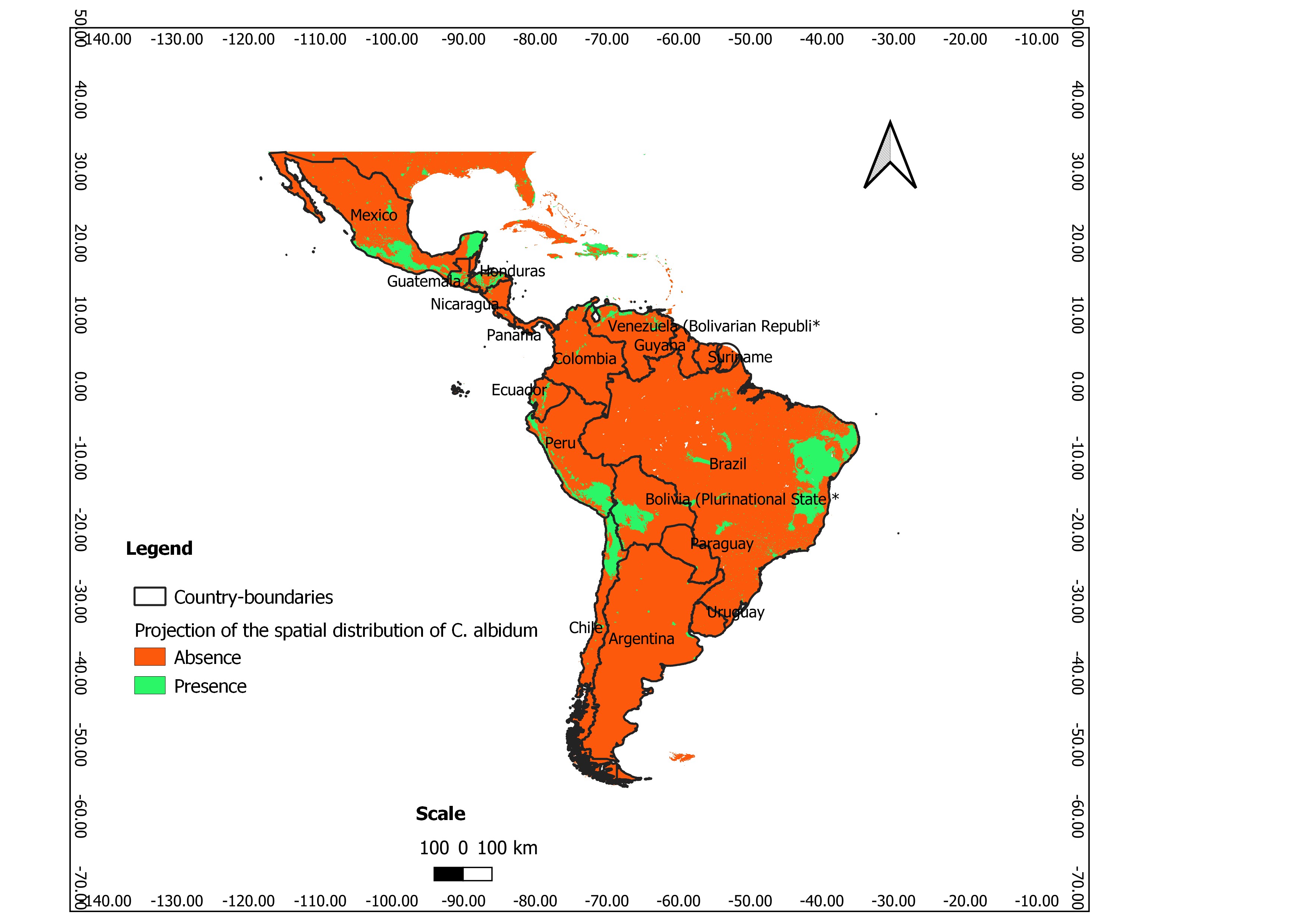

Supplement: Supplementary file 1 — Supplementary Information 1. [file 41598_2023_29048_MOESM1_ESM.zip › GANGLO_Appendices/Appendix_4/Appendix_4b.jpg]

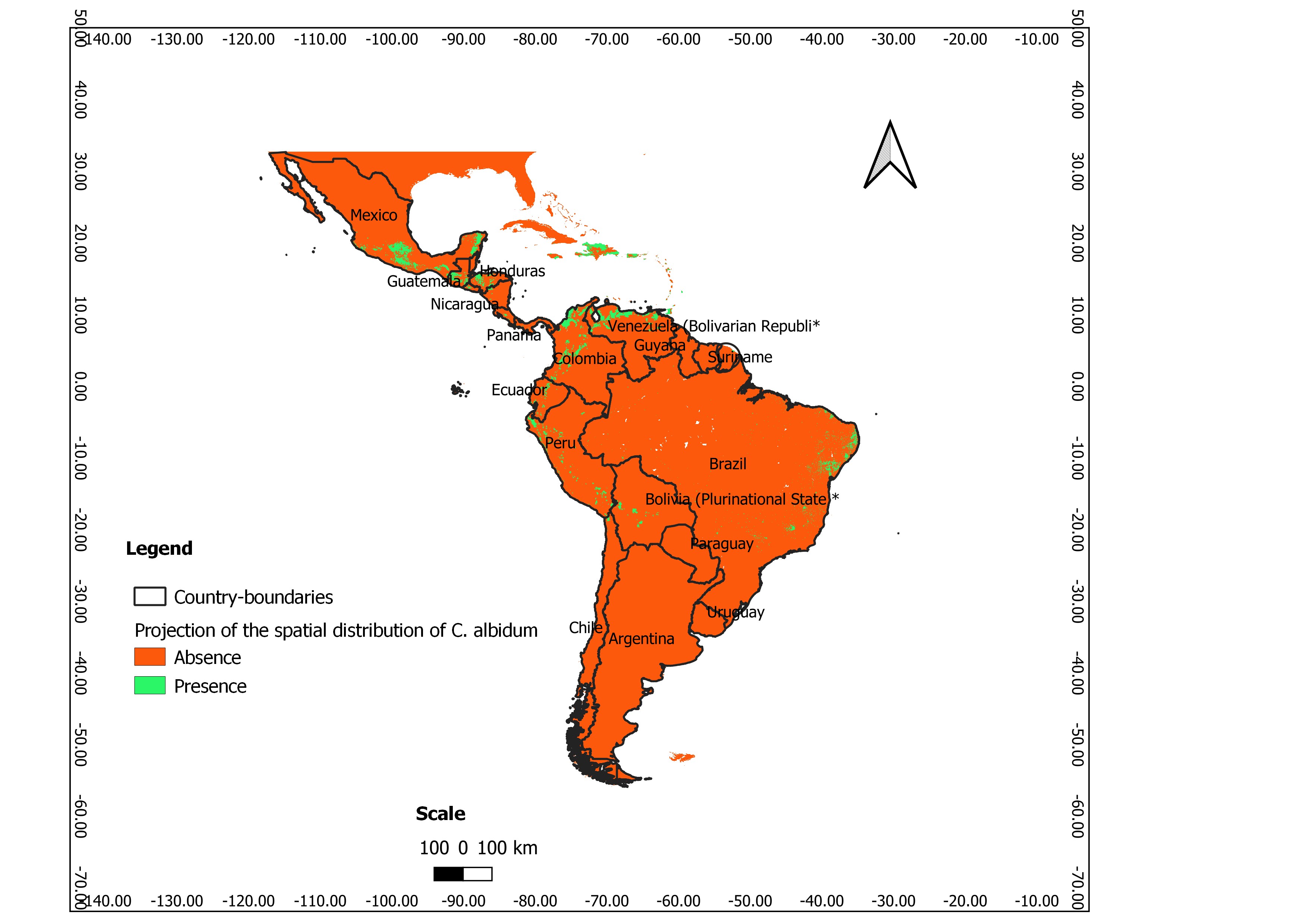

Supplement: Supplementary file 1 — Supplementary Information 1. [file 41598_2023_29048_MOESM1_ESM.zip › GANGLO_Appendices/Appendix_4/Appendix_4a.jpg]

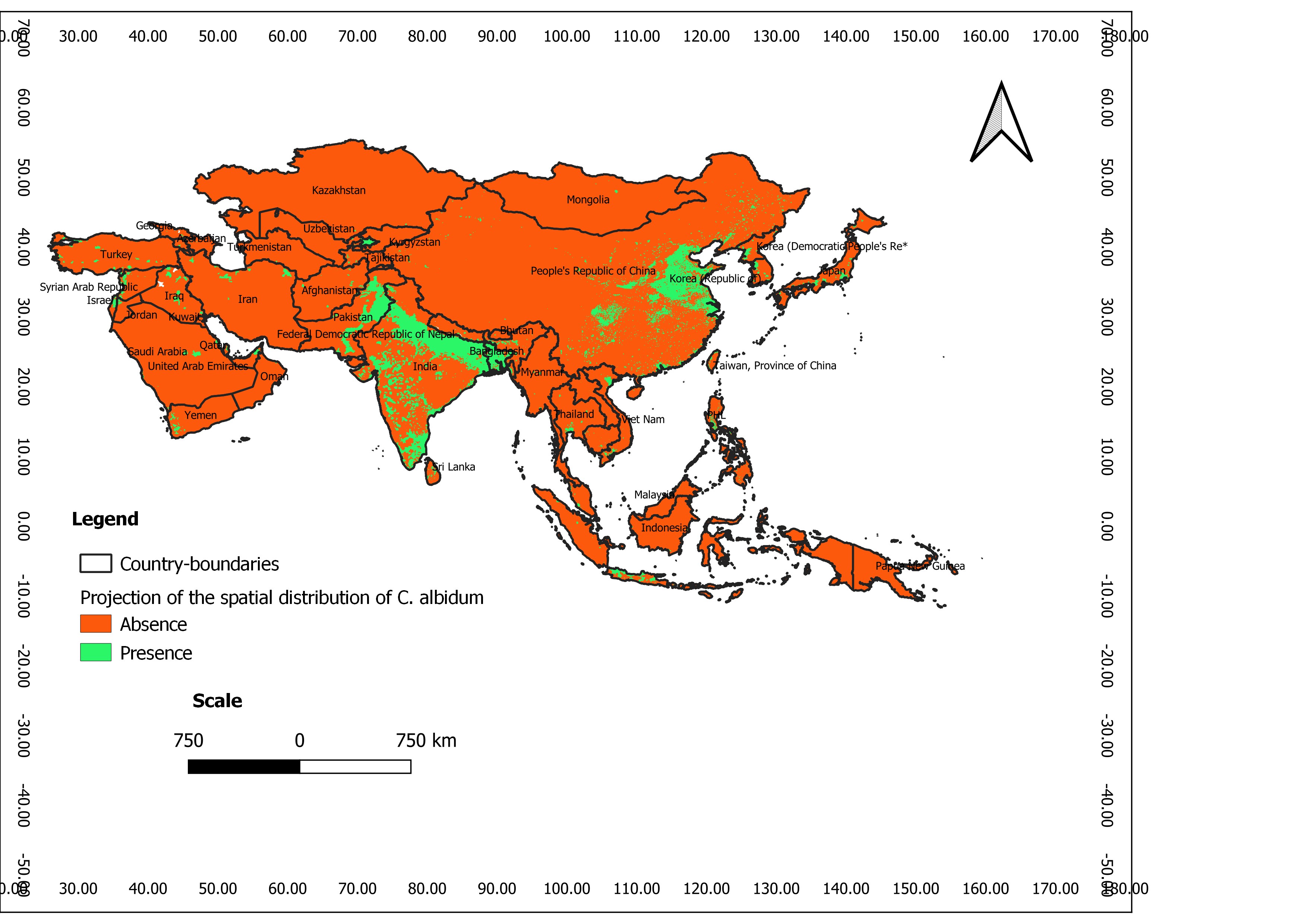

Supplement: Supplementary file 1 — Supplementary Information 1. [file 41598_2023_29048_MOESM1_ESM.zip › GANGLO_Appendices/Appendix_5/Appendix_5a.jpg]

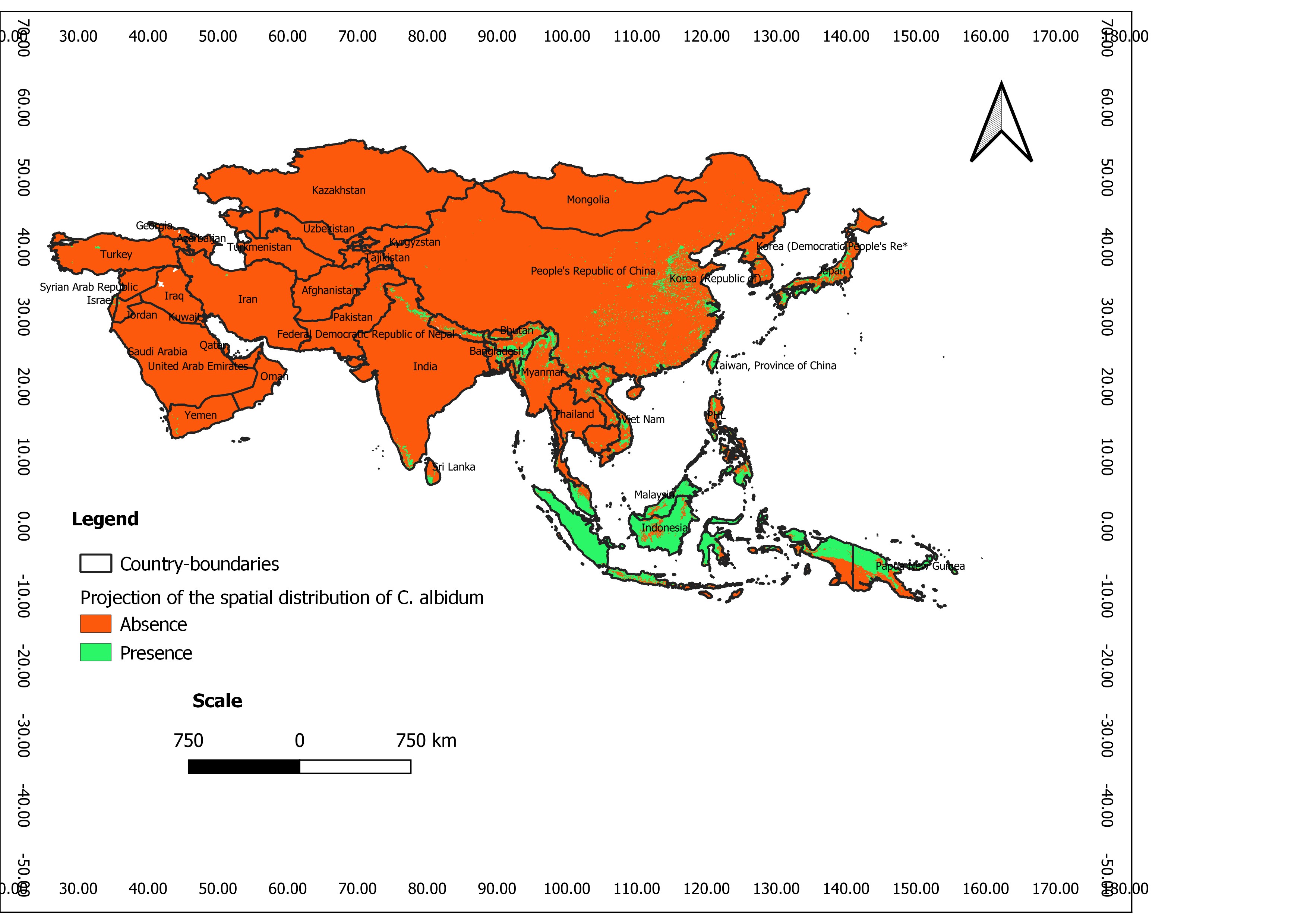

Supplement: Supplementary file 1 — Supplementary Information 1. [file 41598_2023_29048_MOESM1_ESM.zip › GANGLO_Appendices/Appendix_5/Appendix_5b.jpg]
